# In situ APMV-4 vaccination primes systemic response to subtherapeutic dosing of aCTLA-4 in colorectal carcinoma

**DOI:** 10.1136/jitc-2026-015859

**Published:** 2026-09-28

**Authors:** Yonina Bykov, Natalia Alonso Moreda, Naseem Sadek, Gloria Dawodu, Srivatsan Swaminathan, Stephen T Yeung, Daniel Lozano Ojalvo, Javier De Las Rivas, Jeffrey A Tomalka, Adolfo García-Sastre, Sara Cuadrado-Castano

**Affiliations:** 1 Department of Microbiology, Icahn School of Medicine, New York, New York, USA; 2 Bioinformatics and Functional Genomics Group, IBMCC, Salamanca, Spain; 3 IBSAL, Salamanca, Spain; 4 Department of Pathology and Laboratory Medicine, Emory University School of Medicine, Atlanta, Georgia, USA; 5 Emory University School of Medicine, Atlanta, Georgia, USA; 6 Icahn School of Medicine, New York, New York, USA; 7 Division of Infectious Diseases, Weill Cornell Medicine, New York, New York, USA; 8 CIAL, Madrid, Spain; 9 Department of Medicine, Icahn School of Medicine, New York, New York, USA; 10 Department of Pathology, Molecular And Cell-Based Medicine, Icahn School of Medicine, New York, New York, USA

**Keywords:** Colorectal Cancer, Immunotherapy, Oncolytic virus, Solid tumor, Intratumoral

## Abstract

**Background:**

Colorectal carcinoma (CRC) is frequently typified by chronic inflammation and high levels of immunosuppression that render it resistant to immunotherapy. The multimodal immunostimulatory mechanisms of oncolytic virotherapy make it an attractive strategy for priming tumors for immune-checkpoint blockade (ICB). Here, we present an emerging oncolytic virotherapeutic, rAPMV-4, with previously described efficacy in preclinical models of CRC and melanoma.

**Methods:**

We expand on the established efficacy of APMV-4 by characterizing responses to the virotherapy in preclinical single and bilateral MicroSatellite Stable CRC tumor models. To investigate local and systemic effects of the virus, we characterized responses in tumors, draining lymph nodes, and sera using RNA-seq, high-dimensional flow cytometry, and cytokine analysis.

**Results:**

rAPMV-4 induced rapid, yet transient, interferon signaling, and early signatures of antigen presentation and T cell activation. Comparison with a human CRC cohort revealed a transcriptomic shift induced by the virus from a mesenchymal CMS4 to an “immune-high” CMS1 subtype predicted to respond well to ICB. Leveraging these signatures of response and resistance to the virotherapy, we designed a rational combinatorial approach with low-dose anti-CTLA-4, which reversed Treg activation and alleviated CD4 and CD8 dysfunction, leading to more potent systemic tumor elimination.

**Conclusions:**

This work supports the further clinical development of rAPMV-4, either alone or in combination, as an efficacious therapy for CRC.

**WHAT IS ALREADY KNOWN ON THIS TOPIC**

- Colorectal carcinoma, particularly microsatellite stable (MSS) disease, is a major cause of cancer-related morbidity and mortality, with only small subsets of patients demonstrating a long-term response to existing therapeutics. Here, we present a novel oncolytic virotherapy, rAPMV-4, with previously demonstrated efficacy in multiple murine models.

**WHAT THIS STUDY ADDS**

- The study presented here reflects a multidisciplinary and highly integrative effort, combining molecular virology, cancer biology, and systems immunology to investigate virus-tumor-host interactions that underlie the in vivo efficacy of rAPMV-4. Our work identifies broad and potent immunostimulatory capabilities of rAPMV-4 that sensitize MSS colorectal cancers to immune checkpoint blockade.

**HOW THIS STUDY MIGHT AFFECT RESEARCH, PRACTICE OR POLICY**

- By merging comprehensive *in vivo* immune profiling with transcriptomic and *in silico* analyses, we not only delineate the mechanisms underlying rAPMV-4-induced immune activation but also uncover potential biomarkers that may help identify patient subsets most likely to benefit from this therapy in the future.

## Introduction

 Colorectal carcinoma (CRC) represents a disproportionately large fraction of cancer cases worldwide. It was the third most frequently diagnosed cancer type in 2022 and the second leading cause of cancer-related deaths worldwide in the same year,^1^ indicating an urgent need for the development of effective therapeutic strategies. Current treatment standards include surgery, chemotherapy, and T cell-targeted immunotherapy, depending on the stage and microsatellite instability (MSI) status.^2^ While immunotherapy and small molecule inhibitors for MEK and EGFR have gained some favor in recent years, improvements in survival remain minimal and adverse effects high.^3^ Furthermore, these classes of drugs provide benefit to only a subset of patients,^4^ and the immunosuppressive tumor microenvironment (TME) and early metastatic onset tend to be major hurdles to the therapies.^5 6^

Oncolytic virotherapy is an emerging class of cancer immunotherapies that holds much promise due to its ability to modify immunosuppressive TMEs toward immunoreactive TMEs through a multimodal mechanism. Delivery of the viral vector, primarily through intratumoral administration, results in the infection of susceptible cancer cells and recognition of the viral vector by pattern recognition receptors. The subsequent activation of innate antiviral responses and immunogenic cell death^7 8^ leads to production and release of interferons, cytokines, and chemokines that recruit and activate NK cells, T cells, and DCs, polarize monocytes/macrophages toward an M1-like anti-tumor phenotype, and critically, induce *de novo* T cell priming. Indeed, the ability of select virotherapeutics to induce unmasking of tumor-specific and tumor-associated antigens, coupled with the development of antiviral T cells that can be co-opted for antitumoral responses, makes them effective *in situ* tumor therapeutic vaccines.

To date, three genetically modified human DNA-based oncolytic virotherapeutics have achieved clinical approval, although for restricted applications.^9 10^ While these treatments represent a significant advance, they face challenges related to pre-existing host immunity and potential safety concerns. This highlights the growing interest in RNA-based non-human pathogens as highly attractive oncolytic platforms. Their appeal stems primarily from a superior safety profile^11 12^ (due to cytoplasmic replication, lack of genomic integration, and inherent attenuation in humans) and the absence of widespread pre-existing immunity in the human population.^8^

Recently, our group conducted a series of *in vivo* screenings of avian avulaviruses (non-human ssRNA(−) viruses) including Newcastle Disease Virus (NDV) and other avian viruses from the same group. While NDV has been used with some notable successes in preclinical^13–21^ and clinical^22–25^ studies, many patients have historically failed to induce complete responses to *in situ* vaccination with this vector, indicating the necessity of more efficacious approaches. We identified APMV-4 as superior to NDV in preclinical tumor models, inducing a 100% complete remission rate in a syngeneic CT26 CRC tumor model.^26^ It was shown that infection with APMV-4 induced a stronger and more rapid type I IFN response *in vitro* compared with NDV;^26^ however, the host immune responses underlying the increased efficacy of this emergent therapeutic platform have not yet been described. To facilitate studies related to viral biology and to provide flexibility for vector modification and incorporation of therapeutic payloads in future studies, we generated a recombinant infectious clone of our original isolate, rAPMV-4,^26^ employed in our current investigation.

Here, we aimed to preclinically evaluate the potential clinical applicability of APMV-4 in CRC and develop a rational combinatorial treatment approach. For this purpose, we used the CT26.WT CRC tumor model given its (1) remarkable response rate to rAPMV-4, (2) *Kras* mutation status, prevalent in 40%–52% of CRC patients^27^ and associated with increased susceptibility of tumors to RNA viruses,^28–30^ and (3) microsatellite stability (MSS), which is associated with resistance to immunotherapy in CRC patients and presents a significant unmet clinical need.^31^

We evaluated local and systemic responses to the virotherapy by integrating transcriptomic, cellular, and serum cytokine responses throughout the course of treatment of mice-bearing tumors. This systems-based approach allowed us to identify critical mediators of response to rAPMV-4 *in vivo*, the translational relevance and predictive value of which were elucidated in a cohort of 1273 treatment-naive CRC patient samples. Given the high level of heterogeneity and diverse inflammatory signatures characteristic of CRC, we performed our analysis after an initial stratification into previously defined^32^ Consensus Molecular Subtypes (CMS). To our knowledge, this is the first study deconvoluting the CMS-specific risk contribution of virotherapy treatment gene signatures in CRC. Our pipeline allowed us to identify relevant predictors of response to immune-checkpoint blockade (ICB) in human CRC patients also induced by rAPMV-4 in the CRC mouse model, informing a rational design of a combinatorial regimen that reversed resistance to immunotherapy in a model of disseminated disease. All in all, this work presents a systems-based approach for development of a clinical candidate virotherapy with the potential to extend immunotherapy benefit to wider CRC patient populations.

## Materials and methods

### Cell lines, antibodies, other reagents

CT26.WT (ATCC, catalog no. CRL-2638; RRID:CVCL_7254) and Vero cells (ATCC, catalog no. CCL-81; RRID:CVCL_0059) were maintained in RPMI supplemented with 10% FCS, 1% penicillin/streptomycin and normocin. Early passage cells (under passage 20) were used for all experiments. Cancer cells were verified mycoplasma negative by MycoStript-Mycoplasma Detection Kit from InvivoGen (catalog no. rep-mys-50) and underwent IMPACT validation by the Center for Comparative Medicine and Surgery at Icahn School of Medicine at Mt. Sinai. All *in vitro* infections were conducted in Gibco Opti-MEM reduced serum media (ThermoFisher, catalog no. 31985088). APMV-4 chicken antiserum (475-ADV) purchased from the National Veterinary Services Laboratories, US Department of Agriculture (USDA, Ames, Iowa, USA).

### Viruses

rAPMV-4 and rNDV LaSota L289A viral stocks were propagated in 8–9-day-old embryonated chicken eggs for 48–72 hours and clear-purified by ultracentrifugation through a 30% sucrose cushion in NTE buffer. Viruses were then resuspended in Phosphate-buffered saline (PBS) and titered in Vero cells by immunofluorescence.

### Animal studies

8–10-week-old BALB/cJ (RRID:IMSR_JAX:000651) mice were purchased from Jackson Laboratory and were engrafted with 2.5*10^5^ CT26.WT cells in 100 µL RPMI intradermally in the right hind limb for single tumor studies. For bilateral tumor studies, cells were engrafted in the same manner but with 3*10^5^ cells on the right and 1.5*10^5^ cells on the left limb. Treatment of tumor-bearing mice was initiated when tumors reached approximately 45–80 mm^3^ volume. Mice were treated intratumorally with either PBS or 10^7^ PFU APMV-4 in 50 µL PBS every 48 hours for 1–4 total injections. For all studies, tumor volume was measured with calipers every 48 hours, and for all survival studies, mice were euthanized once tumors reached the humane endpoint volume of 1000 mm^3^, exhibited severe ulceration or any sign of severe distress according to IACUC guidelines. Tumor volumes were calculated according to the formula volume=length × width^2^, with length being the longer tumor diameter.

### Flow cytometry

Tumors and tumor-draining lymph nodes were excised at indicated timepoints. Lymph nodes were processed for conventional flow cytometry by mashing through a 70 µm filter, and for spectral flow cytometry by first digesting with 0.5 mg/mL Collagenase D (Roche, catalog no. 11088882001) and 1 µg/mL DNase I (Roche, catalog no. 10104159001) for 30 min at 37°C, then filtering.

Tumors were processed for conventional flow cytometry by chopping into 1 mm^3^ pieces, digesting with Liberase TL (Sigma, catalog no. 5401020001) for 30 min at 37°C, and passing through a 70 µm filter. Tumors were prepared similarly for spectral flow cytometry, but were digested with 0.2 mg/mL Collagenase D and 0.05 mg/mL DNase I for 1 hour at 37°C, then subjected to Percoll purification (Cytiva, catalog no. 17089101) and CD45+cell enrichment (StemCell Technologies, catalog no. 18945) according to manufacturer instructions.

All samples were resuspended in viability dye (see online supplemental material) for 10 min at room temperature. For conventional flow cytometry, tumor and lymph node suspensions were then divided in half for separate staining for the lymphoid and myeloid panels. For tetramer staining only, samples were incubated with tetramer (L(d)/AH1.SPSYVYHQF, PE-conjugated, NIH Tetramer Facility) at a 1:100 dilution for 1 hour at room temperature. Samples prepared for spectral flow cytometry only were preincubated with indicated antibodies for 10 min at 37°C (in one-third of final staining buffer and antibody volumes), before the rest of the extracellular staining mixture was added. All samples underwent extracellular staining with their respective complete antibody mixtures for 40 min at 4^o^C in FACS buffer (2% FBS, 1 mM EDTA in PBS). All samples were washed in FACS buffer, then fixed and permeabilized using the Invitrogen FoxP3 Transcription Factor Staining Buffer Set (Fisher Scientific, catalog no. 50-112-8857) according to manufacturer’s recommendations. See online supplemental material complete list of extracellular and intracellular antibodies used. All samples were washed with permeabilization buffer before resuspension in their respective intracellular antibody mixes for 40 min at 4°C. Samples were washed and resuspended in FACS buffer before acquisition on the 5-laser LSRFortessa X-20 or the 5-laser Cytek Aurora. Data were analyzed using FlowJo software (RRID:SCR_008520) and the unsupflowhelper package in R.

### T cell restimulation

Splenocytes were harvested from bilateral tumor-bearing mice after three doses of the indicated treatment, mashed through 70 µm filters and ACK-lysed before counting and plating in 96-well plates with target cells or peptides. Target cells were infected where indicated with rAPMV-4 8 hours prior and washed with PBS before adding splenocytes at a 1:1 cell ratio. AH1 (tumor-specific) and APHN (APMV-4 specific) peptides were received from GenScript and spiked in at 2.5 uM. APHN was selected based on predictions from NetMHCpan-4.2 and MHCflurry, and validated experimentally.

### Serum cytokine analysis

Blood was collected by submandibular venous puncture and allowed to clot for an hour at room temperature. Samples were then centrifuged at 10 000 g for 10 min to separate serum from whole blood before freezing at −20°C for later use.

Multiplex serum analysis was conducted using the MILLIPLEX mouse cytokine/chemokine multiplex assay kit (Millipore Sigma, catalog no. MCYTOMAG-70K) according to manufacturer instructions and analyzed using the Luminex 200 system and Belysa curve-fitting software.

For soluble CTLA-4, the Mouse CTLA-4 ELISA Kit (ThermoFisher, catalog no. EMCTLA4) was used according to manufacturer recommendations.

### Bulk RNA-seq

Tumors were excised from mice 16 hours after injections 1 and 3, washed in PBS and transferred to homogenization tubes (Benchmark, catalog no. D1032-30) with TRIzol Reagent (ThermoFisher, catalog no. 15596026). Samples were immediately homogenized using the MP FastPrep 24 homogenizer. RNA was extracted with the Direct-zol RNA MiniPrep Plus kit (Zymo Research, catalog no. R2072) according to manufacturer instructions and stored at −80°C. RNA samples were sent for library preparation and bulk RNA sequencing with Azenta. Briefly, RNA underwent PolyA selection before sequencing with the Illumina platform (HiSeq 2×150 bp configuration, 350 million paired-end reads).

QC, trimming, and alignment were performed by Azenta. Downstream analysis was conducted in R. The edgeR package (RRID:SCR_012802) was used for differential gene expression (DEG) analysis, with TMM normalization and glmQLFTest for comparisons. DEGs were selected based on cutoffs of p<0.01 and log2foldchange>1.5. Fgsea (RRID:SCR_020938), enrichplot (RRID:SCR_026996), and clusterProfiler (RRID:SCR_016884) packages were used for gene set enrichment analysis (GSEA), and the gene set variation analysis (GSVA) package (RRID:SCR_021058) was used for GSVA. Significant pathways were selected from GSEA based on a cut-off of p<0.05. ClueGo (RRID:SCR_005748) was used for pathway network analysis. Barplots and volcano plots were generated using ggplot2 (RRID:SCR_014601) and heatmaps using pheatmap (RRID:SCR_016418).

### Human transcriptome analysis

We analyzed a cohort of 1273 primary tumors from CRC patients containing full transcriptomic profiling and phenotypic information.^33^ R was used for all statistical and computational analyses. The integrated and normalized cohort of CRC tumors was filtered to include a subset of 849 individuals with available overall survival data,^33^ and assigned to CRC CMS^32^ using the *CMScaller* tool (RRID:SCR_027275).^34^

We used the org.Mm.eg.db (RRID:SCR_023488) and org.Hs.eg.db (RRID:SCR_024739) libraries to identify human orthologs. Principal Component Analysis (PCA) was performed using *FactoMineR* (RRID:SCR_014602) and *factoextra* (RRID:SCR_016692) libraries. Functional enrichment analysis was conducted with the *clusterProfiler* (RRID:SCR_016884) package, considering Gene Ontology (GO) terms and using an adjusted p value of 0.05 as the threshold in all cases. The overlap of the functions was presented using a Venn diagram of the GO terms, which identifies the significant biological functions common between the human CMS subtypes and the murine samples. Multivariate risk analysis and univariate survival analysis were performed using the ASURI R package.^35^

### In vivo viral replication

For detection of infectious viral particles, tumors were excised at the indicated timepoint after a single intratumoral dose with rAPMV-4 (10^7^ PFU in PBS). Samples were immediately homogenized in PBS using the MP FastPrep 24 homogenizer, and frozen at –80°C. Thawed samples were clarified by centrifugation, and supernatants used to infect Vero cells. Infection, immunofluorescence, and viral titer calculations were performed as described previously.^26^

For measurement of viral mRNA, tumors and draining lymph nodes (dLNs) were excised at the indicated timepoints, rinsed in PBS, and immediately homogenized as described for tumors used for bulk RNAseq analysis. qPCR analysis was performed as described previously.^26^

### Statistical analysis

Analysis was performed using GraphPad Prism 10 (RRID:SCR_002798) and the rstatix package (RRID:SCR_021240) in R. One-way analysis of variance (ANOVA) was used to compare multiple groups with a single independent variable, and two-way ANOVA for two independent variables. Survival analyses were conducted using the log-rank (Mantel-Cox) test, with statistical analysis performed using the Kaplan-Meier method. p>0.05 ns, p<0.05 *, p<0.01 **, p<0.001; ***, p<0.0001 ****.

## Results

### rAPMV-4 monotherapy controls tumor growth and induces complete responses in syngeneic murine colorectal carcinoma models

We previously demonstrated that four intratumoral administrations of rAPMV-4 induce complete tumor regression and protection against rechallenge in up to 100% of treated animals in the single tumor CT26.WT model. Based on this, we proceeded to evaluate the efficacy of rAPMV-4 in a bilateral tumor model (figure 1A), used as a surrogate for multicentric or multifocal tumor dissemination. Indeed, rAPMV-4 as a single agent induced a 57% survival rate (figure 1B), demonstrating control of both treated (figure 1C) and contralateral tumors (figure 1D). Furthermore, the monotherapy induced an abscopal effect, as evidenced by its ability to delay growth and induce complete responses in approximately 50% of contralateral tumors (figure 1D).

**Figure 1:**
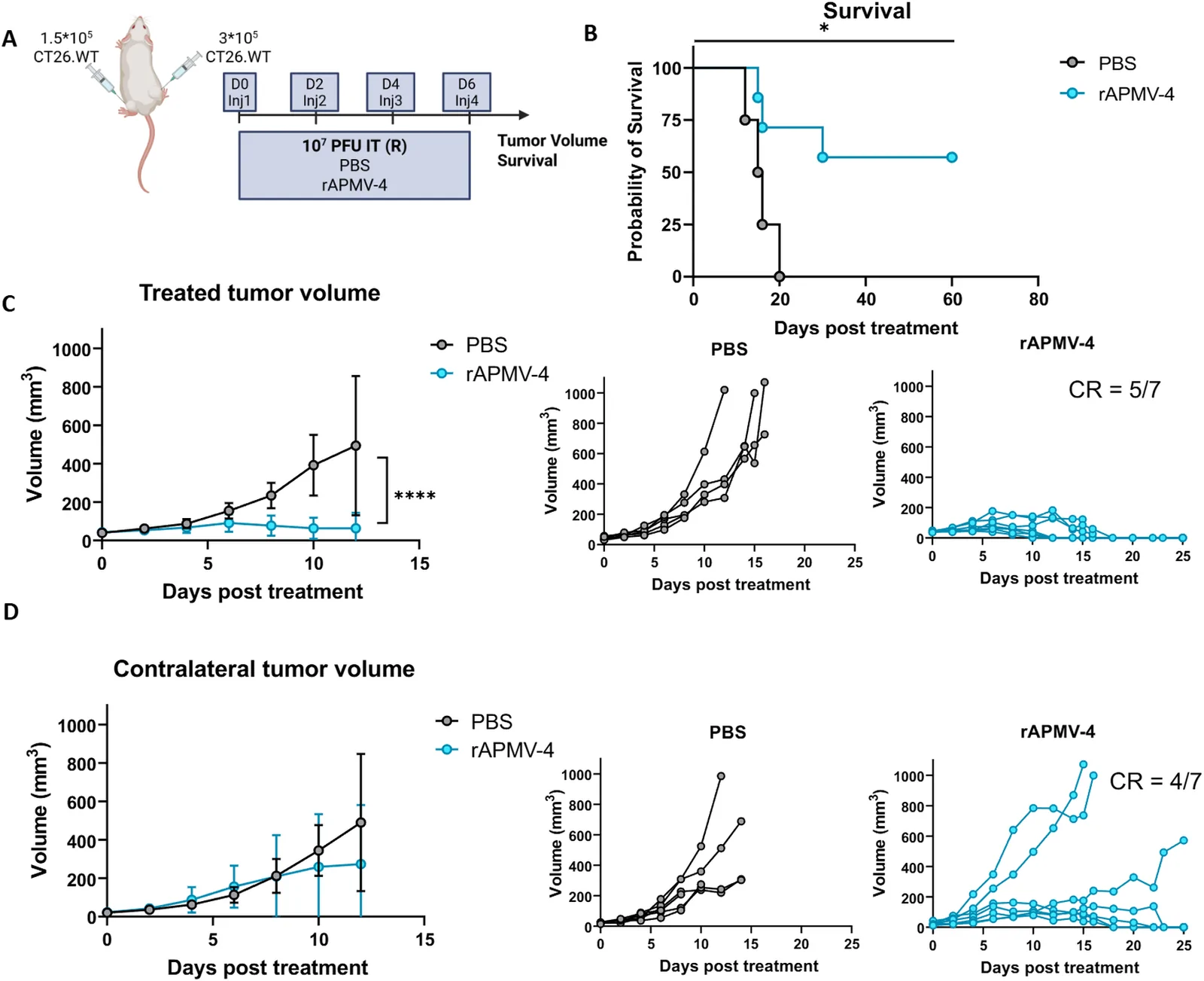
APMV-4 monotherapy induces tumor regression and abscopal effect in CT26.WT model. (**A**) In vivo treatment schematic for the bilateral tumor model in (B–D). (B) Kaplan-Meier survival analysis, with p values calculated using log-rank (Mantel-Cox) statistical analysis. (**C**) Average treated tumor volumes±SEM, with statistical analysis shown from two-way ANOVA at the time of first death (left) and individual treated tumor growth curves of phosphate-buffered Saline (PBS)-treated mice (middle) and APMV-4-treated mice (right). (**D**) Average contralateral (ie, left, or untreated) tumor volumes ±SEM, with statistical analysis shown from two-way ANOVA at the time of first death (left) and individual contralateral tumor growth curves of PBS-treated mice (middle) and APMV-4-treated mice (right). N=5–8 mice per group, *p<0.05; ****p<0.0001; ns, non-significant. ANOVA, analysis of variance; PBS, phosphate-buffered saline; PFU, plaque formation units; CR, complete response.

### rAPMV-4 monotherapy promotes rapid induction of antiviral inflammatory programs and sustained T cell mobilization

Given the efficacy of the rAPMV-4 monotherapy in controlling tumor growth in our murine colorectal carcinoma models, we aimed to understand how intratumoral administration of the virus alters the TME and systemic host immune responses (figures 2 and 3, online supplemental figures S1 and S2and online supplemental table 3). To this end, we conducted bulk RNAseq, flow cytometry, and multiplex serum cytokine analysis as depicted in figure 2A. Pathway network analysis of top DEGs in tumors treated with rAPMV-4 versus PBS control tumors revealed widespread antiviral responses such as type I IFN induction (with leading edge genes including known interferon-stimulated genes such as *Isg15, Oas1,* and *Ifit1*) and cytokine production, along with upregulation of antigen presentation processes (primarily through class 1, which is critical for antigen presentation to CD8 T cells) (figure 2B, online supplemental table 2) after just a single injection, which was also confirmed on the individual gene level (figure 2C). Given the upregulation of antigen presentation pathways with the virotherapy, we wanted to verify whether this also correlated with improved T cell function. Indeed, GSEA analysis identified upregulation of T cell cytotoxicity, migration, and activation pathways (figure 2D), all of which are critical for initiating proper spatiotemporal placement of T cells for induction of antitumor immunity. These transcriptional signatures translated to therapeutic outcomes, as one injection of rAPMV-4 administered in the single tumor model (figure 2F) was sufficient to induce a 38% survival rate (figure 2G) and slowed tumor growth (figure 2H).

**Figure 2:**
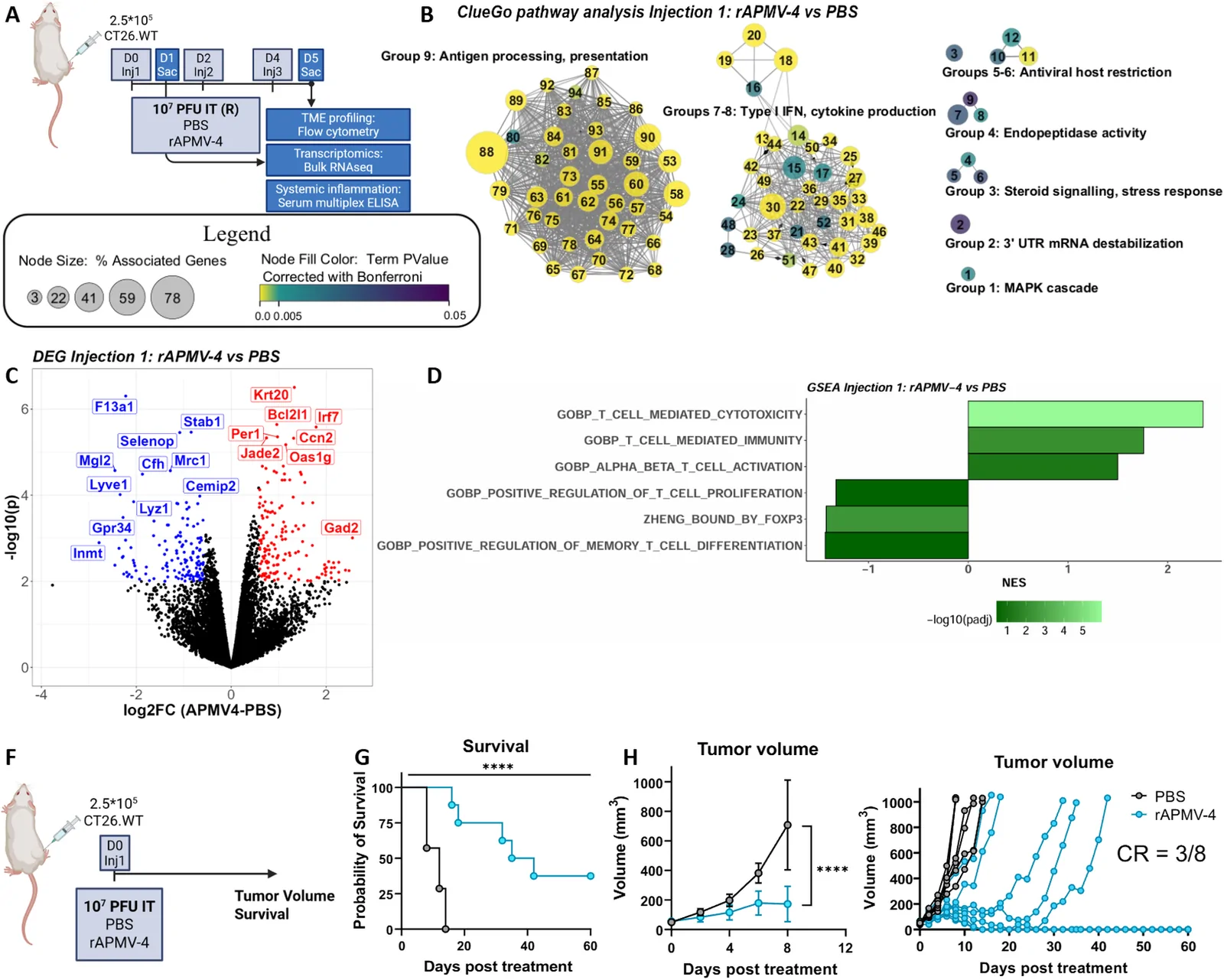
Single dose of rAPMV-4 is sufficient to induce antiviral inflammatory programs and T cell immunity signatures, resulting in control of tumor growth. (**A**) In vivo experimental schematic for (B–D). (B–D), Bulk RNAseq analysis after injection 1 only: (**B**) ClueGo network analysis of top 100 genes upregulated in rAPMV-4 versus PBS-treated tumors after injection 1. (**C**) Volcano plot representing top differentially regulated genes in APMV-4 versus PBS-treated tumors after injection 1. (**D**) Bar plot representing significant (p<0.05) GSEA results filtered on T cell-specific pathways enriched in rAPMV-4 versus PBS-treated tumors after injection 1. (**F**) In vivo treatment schematic for (G–H). (G) Kaplan-Meier survival analysis, with p values calculated using log-rank (Mantel-Cox) statistical analysis. (**H**) Average tumor volumes±SEM, with statistical analysis shown from two-way ANOVA at time of first death (left) and tumor growth curves of individual animals (right). N=3 or 5 animals per group, ****p<0.0001; ns, non-significant. ANOVA, analysis of variance; PBS, phosphate-buffered saline; PFU, plaque formation units; CR, complete response; DEG, differentially expressed gene; GSEA, gene set enrichment analysis; TME, tumor microenvironment.

**Figure 3:**
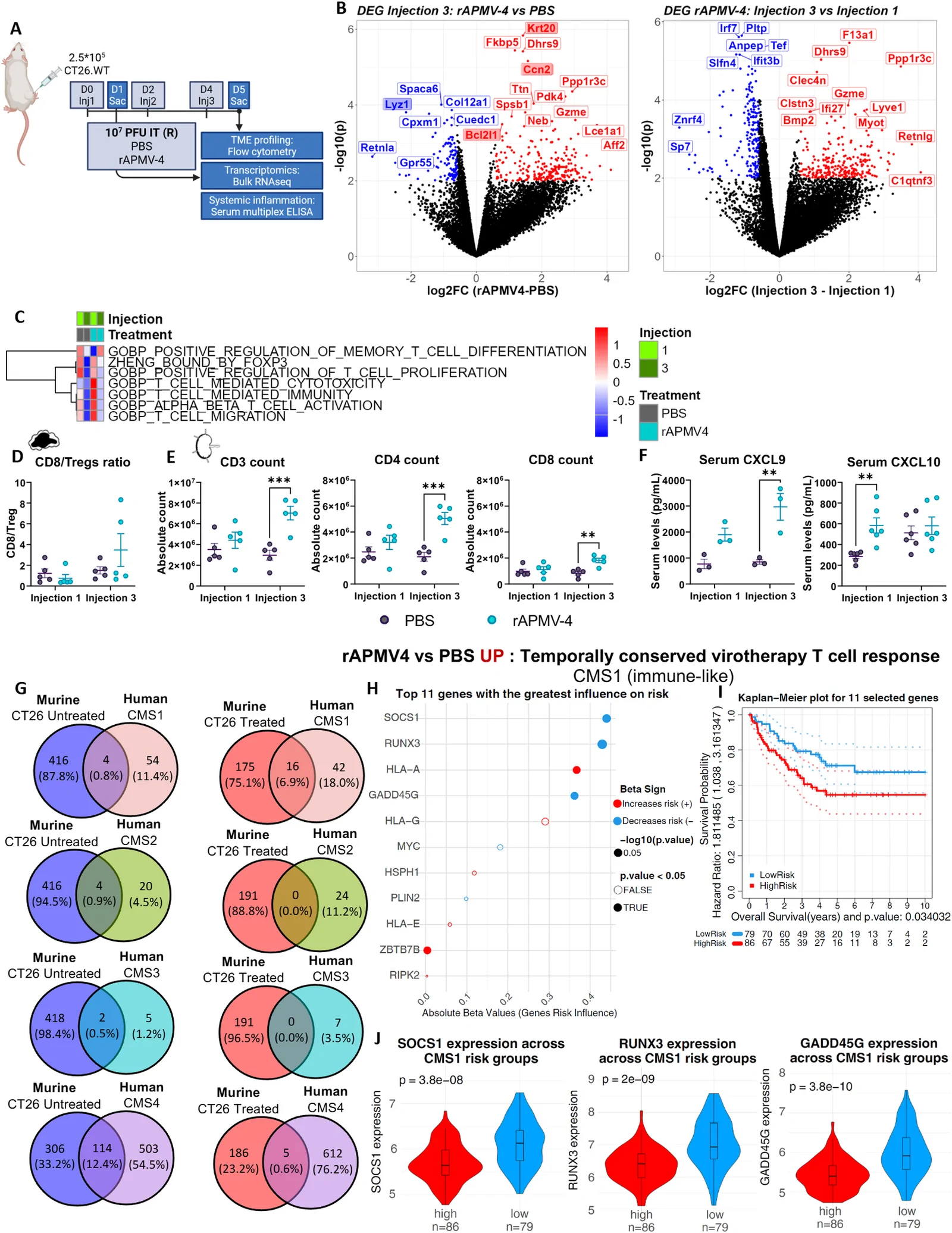
(A) In vivo experimental schematic for (B–F). (B–C) Bulk RNAseq analysis including injections 1 and 3. (**B**) Volcano plot representing top differentially regulated genes in tumors treated three times with rAPMV-4 versus PBS-treated tumors (left) and in rAPMV-4-treated tumors after 3 injections versus 1 injection. Shaded labels indicate DEGs conserved between injections 1 and 3. (**C**) Heat map depicting GSVA for rAPMV-4 and PBS treatment groups after injections 1 and 3 for pathways from 2D. (**D**) Flow cytometric analysis of tumors demonstrating CD8/Treg ratios after 1 or 3 injections with PBS or rAPMV-4. (**E**) Flow cytometric analysis of dLNs depicting T cell frequencies after 1 or 3 injections with PBS or rAPMV-4. (**F**) Luminex serum analysis showing CXCL9 and CXCL10 concentrations after 1 or 3 injections with PBS or rAPMV-4. (**G**) Venn diagrams representing shared GO biological pathways between human primary CRC tumors divided by CMS type and CT26 untreated (left) or treated (right) murine tumors. (**H**) Bubble plot highlighting top genes with the greatest influence on CMS1 patient risk derived from murine T cell gene set enriched in response to rAPMV-4. (**I**) Kaplan-Meier plot for multivariate analysis for CMS1 patient risk groups generated through multivariate analysis of input genes from (H). (**J**) Violin plots for relative expression of selected top genes with influence on risk from (H, I), divided by risk group. For (**B–F**) N=5–8 mice per group, **p<0.01; ***p<0.001; ns, non-significant. CRC, colorectal carcinoma; PBS, phosphate-buffered saline; PFU, plaque formation units; DEG, differentially expressed gene; dLNs, draining lymph nodes; GSVA, gene set variation analysis; TME, tumor microenvironment.

Repeated viral treatment (as detailed in figure 3A) upregulated granzymes as well as *Lyve1* (figure 3B), suggesting further enhancement of T cell effector function and lymphangiogenesis to support T cell tumor infiltration. GSVA confirmed general conservation of multiple T cell-related pathways when comparing timepoint-matched APMV-4 versus PBS-treated tumors, with a notable shift toward T cell proliferation and memory T cell differentiation after 3 injections (figure 3C). While memory T cell differentiation also appeared enriched in untreated samples analyzed after a single injection, it is likely that any memory T cells in this context were dysfunctional as evidenced by the lack of cancer-specific T cell responses in treatment-naive animals (online supplemental figure S11e–h). We confirmed our RNAseq findings by flow cytometry at matched timepoints, highlighting a trend toward increased CD8/Treg ratios in the tumors of a subset of treated animals (figure 3D), as well as increased CD4 and CD8 T cell counts in dLNs (figure 3E). Tumor-bearing mice treated with rAPMV-4 also had increased serum levels of CXCL9 and CXCL10 (figure 3F), suggesting systemic T cell mobilization.

### Translational applicability of murine transcriptomic signatures to human CRC

Having identified the transcriptomic and cellular signatures of response to rAPMV-4 in our murine model, we aimed to evaluate their translational relevance (figure 3G–J, online supplemental figures S3–S6). Leveraging a cohort of treatment-naive human CRC primary tumors stratified by CMS type, we conducted pathway comparison between each of the four CMS types and our murine tumors. Untreated CT26 tumors were most similar to CMS4 CRC, sharing 114 GO pathways representing angiogenesis, stemness, and extracellular matrix organization (figure 3G, online supplemental figure S3c). In contrast, rAPMV-4 treated murine tumors were most similar to CMS1, sharing 16 pathways representing antiviral responses (figure 3G, online supplemental figure S3d). To evaluate the clinical relevance of T cell-related pathways enriched with rAPMV-4 treatment (figures 2D and 3C), we assembled a temporally conserved set of leading edge genes upregulated in virus-treated tumors at injections 1 and 3. Human orthologs of these genes were used as input for multivariate risk analysis for each CMS type, effectively stratifying CMS1 patients into low-risk and high-risk groups, with most significant genes having a negative risk influence (figure 3H). Low-risk and high-risk CMS1 groups exhibited a significant difference in survival (figure 3I), suggesting a correlation between higher expression of negative risk contributors and improved outcomes (which we verified by univariate survival analysis in online supplemental figure S5a–d). The greatest contributors to reduced risk were SOCS1, RUNX3, GADD45, confirmed as significant in our model by comparing their expression in high-risk versus low-risk groups (figure 3J). All three genes have tumor suppressor functions in multiple cancer types, with the first two strongly linked to T cell activation and maturation.

### Low-dose aCTLA-4 coadministration with rAPMV-4 boosts antitumor effect of monotherapies

Our analysis broadly linked response to rAPMV-4 with T cell activation and infiltration into treated tumors (with curated response signatures correlating with improved survival in human CRC cohorts), yet we observed several indicators of resistance to therapy demonstrating a need for a combinatorial approach to boost systemic efficacy: (1) a number of tumors refractory to rAPMV-4 in the contralateral site in the bilateral tumor model (figure 1D), and (2) development of Treg transcriptional (figure 3C) and cellular (figure 3D) signatures in treated tumors after repeat dosing. Given some existing precedent of CTLA-4 blockade in CRC for the reversal of effector T cell dysfunction and concomitant Treg depletion,^36^ we designed a treatment approach using a low dose (2 mg/kg) of CTLA-4 blocking antibody (aCTLA-4) administered systemically as depicted in figure 4A. While both monotherapies induced a 50% complete response rate, the combinatorial regimen induced a 100% complete response rate in both tumor sites (figure 4B,C). Comparison with the clinical candidate strain NDV LaSota L289A demonstrated a synergistic effect between rAPMV-4+aCTLA-4 but not between NDV+aCTLA-4 (online supplemental figure S8a–d), suggesting superior utility of rAPMV-4 in this tumor model.

**Figure 4:**
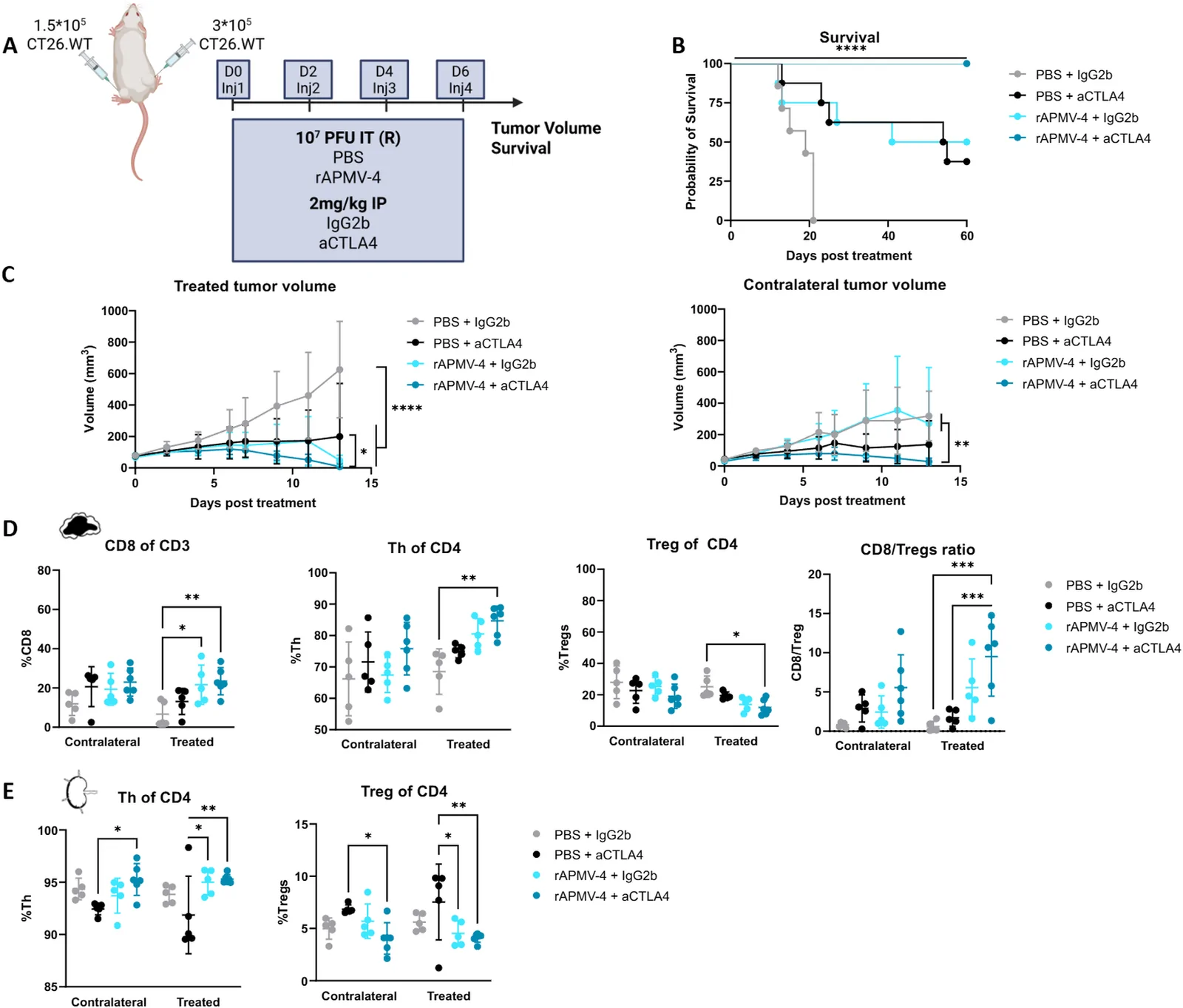
Low-dose aCTLA-4 coadministration with APMV-4 boosts antitumor effect of monotherapies. (**A**) In vivo treatment schematic for the bilateral tumor model in (**B, C**). N=5–8 animals per group. (**B**) Kaplan-Meier survival analysis, with p values calculated using log-rank (Mantel-Cox) statistical analysis. (**C**) Average treated (injected) tumor volumes±SEM (left) and average contralateral (untreated) tumor volumes ±SEM (right) for each treatment group, with statistical analysis shown from two-way anova at the time of first death. (**D**) Flow cytometric analysis of T cell frequencies in treated and contralateral tumors 16 hours post injection 3. (**E**) Flow cytometric analysis of T cell frequencies in treated and contralateral dLNs 16 hours post injection 3. *p<0.05; **p<0.01; ***p<0.001; ****p<0.0001; ns, nonsignificant. dLNs, draining lymph nodes. PBS, phosphate-buffered saline; PFU, plaque formation units.

Conventional flow cytometric analysis of treated and contralateral tumors revealed significantly increased frequency of CD8 T cells and Th cells, and reduced Treg frequency in treated tumors in response to rAPMV-4 virotherapy, with all trends boosted by coadministration with aCTLA-4 (figure 4D; gating strategy in online supplemental figure S7). No significant changes in T cell population frequencies were observed in the contralateral tumors; however, animals treated with rAPMV-4+aCTLA-4 had the highest CD8/Treg ratios of all the treatment groups, with two out of six tumors responding particularly strongly to the therapy (figure 4D). In the dLNs, the combination therapy induced the greatest increase in Th and decrease in Treg proportions in treated and contralateral sites (figure 4E).

### rAPMV-4 combined with aCTLA-4 prevents T cell exhaustion in treated and contralateral tdLN and tumors

To further investigate immunophenotypic changes underlying the response to combinatorial therapy, we performed spectral flow cytometric profiling of treated and contralateral tumors and their dLNs (figures 5–7, online supplemental figures S9–S11a–c, S12) as depicted in the experimental schematic in figure 5A. Unsupervised analysis of dLNs revealed broad remodeling of the T cell compartment (figure 5B) in response to single and combinatorial treatments. The greatest T cell expansion occurred in the treated dLNs in response to combination therapy, as seen by supervised analysis (figure 5C). rAPMV-4, particularly in combination with aCTLA-4, drove a significant increase in the proportion of Th1 and Tfh in the treated dLN. aCTLA-4 alone or in combination with rAPMV-4 boosted Th1 and Tfh frequencies in the contralateral dLN, though to a lesser degree than in the treated side (figure 5D). Th2 and Th17 portions increased only slightly or were unchanged by treatment (online supplemental figure S10b).

**Figure 5:**
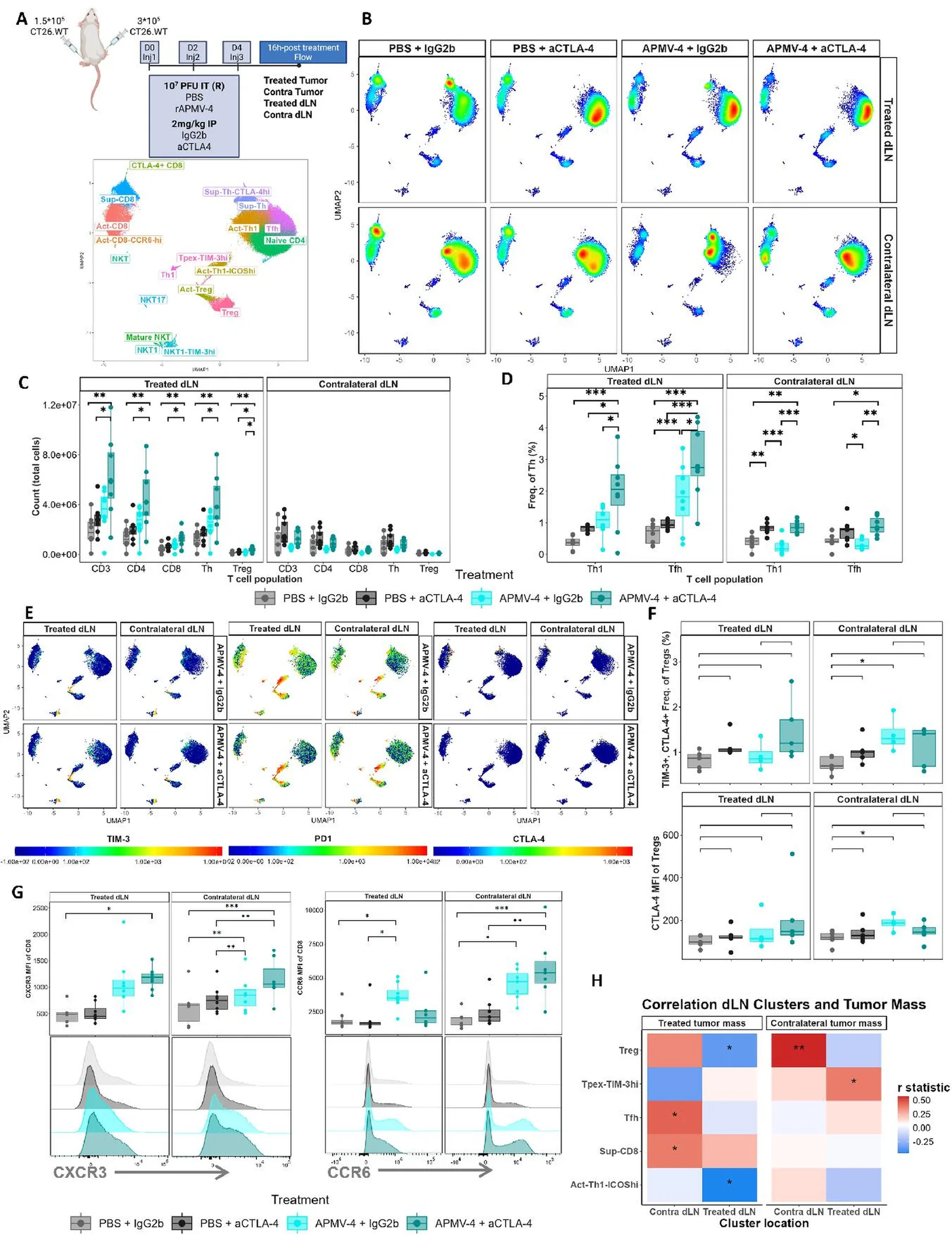
rAPMV-4 combined with low-dose aCTLA-4 prevents T cell exhaustion in treated and contralateral tdLN. (**A**) In vivo experimental schematic for (B–H). (B) Uniform Manifold Approximation and Projection (UMAP) plot depicting cluster labels for all dLN samples (left) and UMAP density plots of CD3+compartment separated by treatment group and dLN location (right). (**C**) T cell frequencies in treated and contralateral dLNs identified by supervised analysis. (**D**) Th frequencies in treated and contralateral dLNs identified by supervised analysis. (**E**) TIM-3 (left), PD1 (middle), and CTLA-4 (right) heatplots visualized on UMAP projections of treated and contralateral dLN from mice treated with either APMV-4 or APMV-4+aCTLA-4. (**F**) TIM-3+, CTLA-4+frequency (top) and CTLA-4 Mean Florescence Intensity (MFI) of Tregs (bottom) obtained from supervised analysis (N=4–5 animals per treatment group). (**G**) CXCR3 (top left) and CCR6 (top right) MFI of CD8 from all animals with representative histograms for one animal per treatment group for both tissue sites shown below. (**H**) Pearson correlation analysis of clusters identified by unsupervised analysis of treated and contralateral (contra) dLN and treated and contralateral tumor masses, with color representing the r statistic. Only clusters that yielded a significant p value for at least one tissue site are shown here. N=8 animals per treatment group (unless otherwise indicated), *p<0.05; **p<0.01; ***p<0.001; ns, nonsignificant. dLNs, draining lymph nodes.

**Figure 6:**
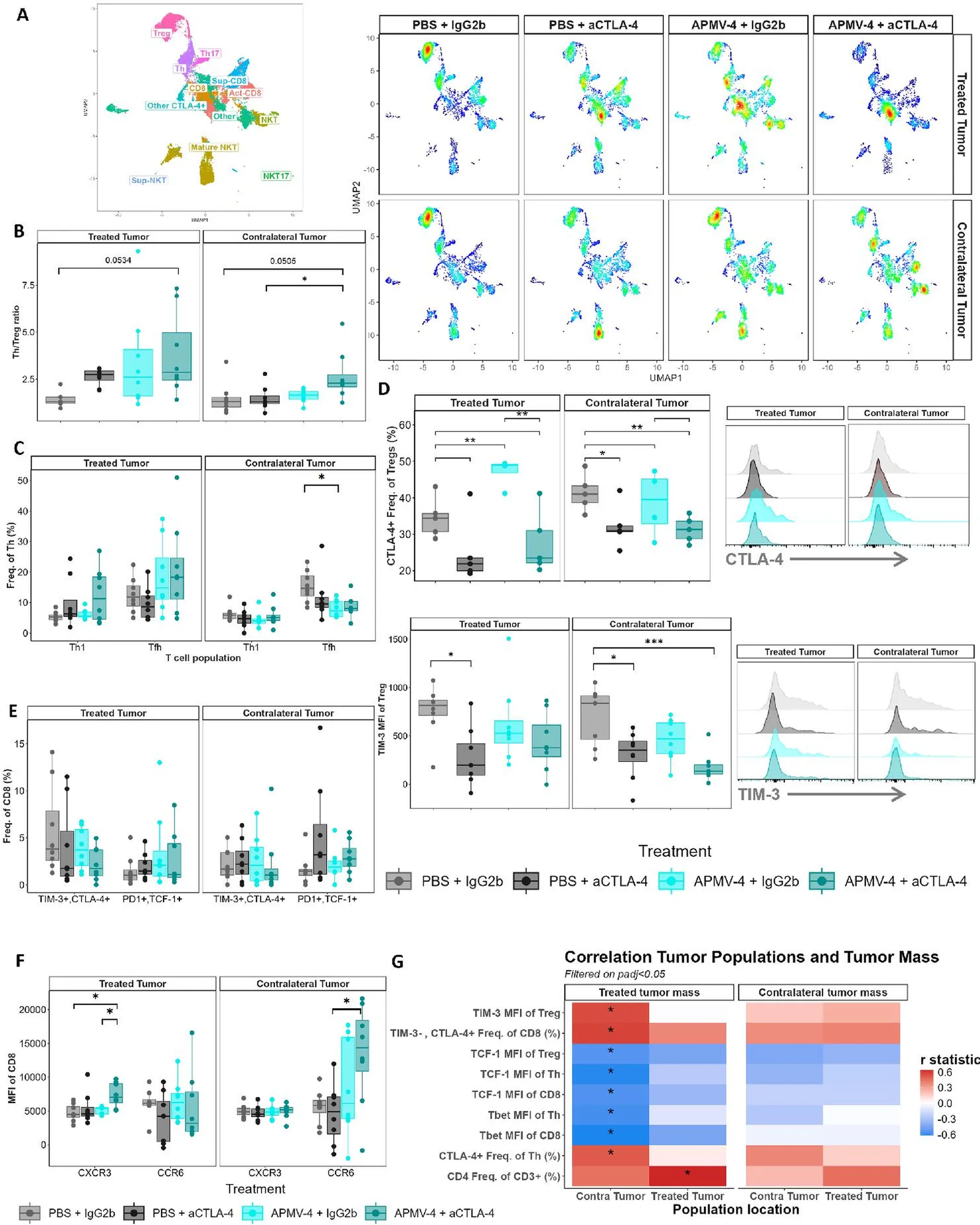
rAPMV-4 combined with low-dose aCTLA-4 induces T cell recruitment into treated and contralateral tumors. (**A**) Manifold Approximation and Projection (UMAP) plot depicting cluster labels for all tumor samples (left) and UMAP density plots of CD3+ compartment separated by treatment group and tumor location (right). (**B**) Th/Treg ratios in treated and contralateral tumors as determined by supervised analysis. (**C**) Th frequencies in treated and contralateral tumors identified by supervised analysis. (**D**) CTLA-4 frequency (top left, N=4–5 animals per treatment group) and TIM-3 MFI (bottom left) of Tregs from all animals with representative histograms for one animal per treatment group for both tissue sites shown to the right. (**E**) TIM-3+, CTLA-4+ and PD1+, TCF-1+ frequencies of CD8 T cells in treated and contralateral tumors identified by supervised analysis. (**F**) CXCR3 and CCR6 MFIs of CD8 T cells identified by supervised analysis of treated and contralateral tumors. (**G**) Pearson correlation analysis of populations identified by supervised analysis of treated and contralateral (contra) tumors and treated and contralateral tumor masses, with color representing the r statistic. Only clusters that yielded a significant padj value for at least one tissue site are shown. N=8 animals per treatment group (unless otherwise indicated), *p<0.05; **p<0.01; ***p<0.001; ****p<0.0001; ns, non-significant; in (G) the padj values are represented. PBS, phosphate-buffered saline; MFI, Mean Florescence Intensity.

**Figure 7:**
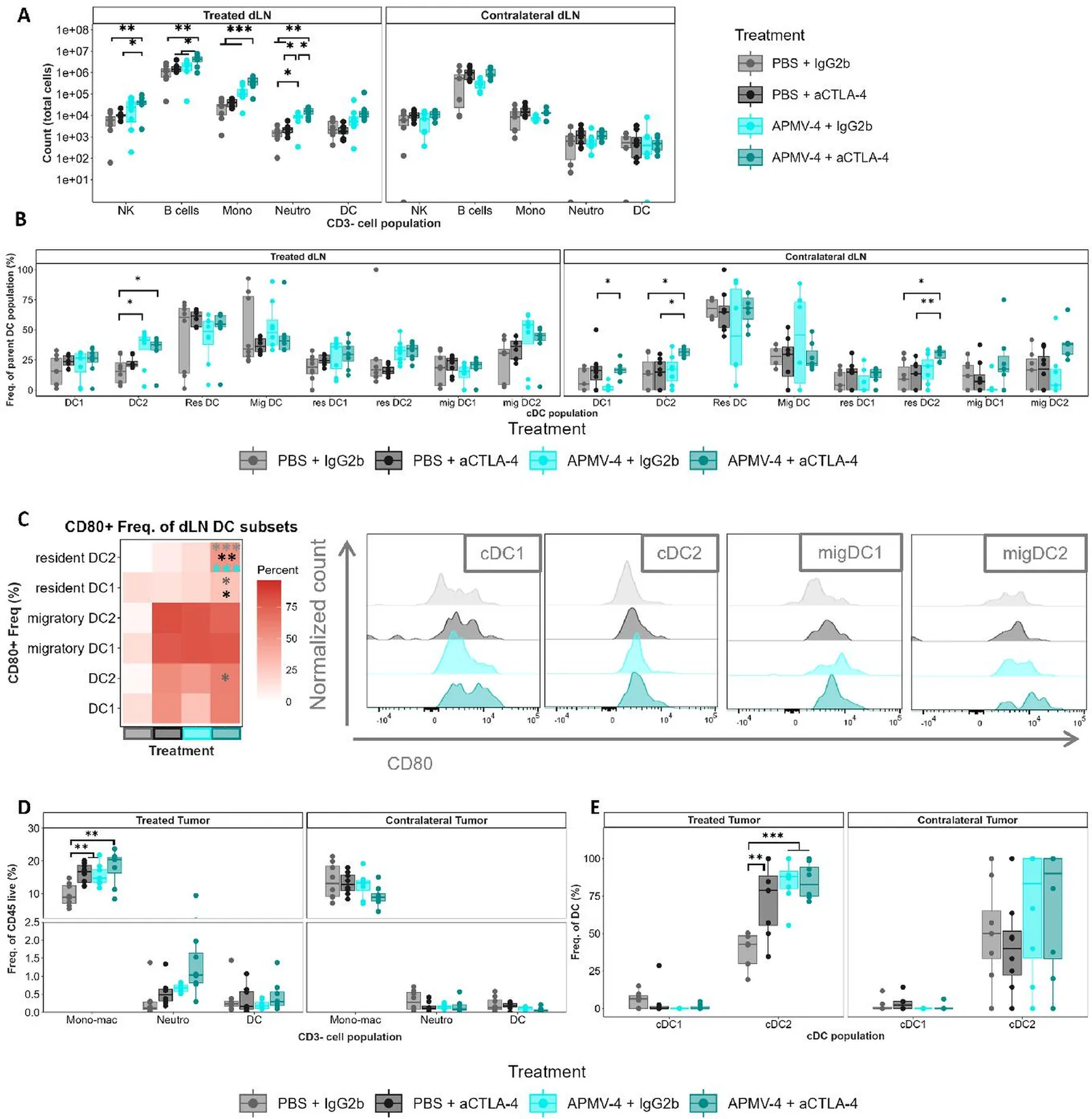
Innate immune recruitment and activation in response to rAPMV-4 and aCTLA-4 in tumors and dLNs. (**A**) Total counts of major CD3− immune populations in treated and contralateral dLNs. (**B**) Frequencies of DC subsets of their respective parent populations in treated and contralateral dLNs. (**C**) Frequencies of CD80+ cells in indicated treated dLN DC subsets summarized in a heatmap (left) and in representative histograms (right) for one animal per treatment group. (**D**) Frequencies of major CD3− immune populations in treated and contralateral tumors. (**E**) Frequencies of DC1 and DC2 of total DC in treated and contralateral tumors. N=8 animals per treatment group, *p<0.05; **p<0.01; ***p<0.001; ****p<0.0001; ns, non-significant. dLN, draining lymph node.

To assess T cell functionality, we used three canonical markers of T cell dysfunction, CTLA-4, TIM-3, and PD-1. dLNs of mice treated with rAPMV-4 maintained expression of all three markers on Th and Treg clusters, while the supplementation of the virotherapy with aCTLA-4 induced a downward trend in their expression in all major T cell populations (figure 5E,F, online supplemental figure S10a) in both sites. Concurrently with the reversal of effector T cell suppression and Treg activation, the combinatorial therapy increased CXCR3 (and induced a notable, though not statistically significant trend of increased CXCR5) expression on CD8 T cells in treated and contralateral dLNs, as well as CCR6 in the contralateral dLN (figure 5G, online supplemental figure S10c). These changes are consistent with CD8 activation and migratory capacity that are critical for trafficking to the tumor site. While aCTLA-4 monotherapy did not generally alter chemokine expression on dLN CD8 T cells, rAPMV-4 monotherapy did induce upregulation, particularly in the treated dLN, though not to the same level as the combination therapy. These shifts from exhausted to activated CD8 and CD4 phenotypes induced by the combination therapy were also broadly observed using unsupervised analysis (figure 5B).

To validate the impact of dLN T cell clusters identified by unsupervised analysis on tumor growth, we conducted Pearson correlation analysis for cluster frequency in dLNs and treated and contralateral tumor masses. The most significant positive correlation was found between Treg frequency in the contralateral dLN and contralateral tumor mass, with notable positive correlations between suppressed CD8 and precursor exhausted T cell (Tpex) clusters in the contralateral and treated dLNs and their respective tumors (figure 5H). The most significant negative correlation was between treated dLN ICOS^hi^ Th1 and treated tumor mass, with additional negative correlations between treated dLN activated CCR6^hi^ CD8 T cells and contralateral tumor mass (figure 5H, online supplemental figure S10d). The strongest correlation being between contralateral dLN Treg frequency and contralateral tumor mass highlights the central role of Tregs in resistance to virotherapy (particularly in distal lesions) in this tumor model and the potential utility of specific Treg targeting in future combinatorial approaches.

As in the dLNs, unsupervised analysis of tumors revealed broad T cell remodeling in response to rAPMV-4 and aCTLA-4. Tregs and exhausted CD8 clusters (expressing CTLA-4, TIM-3, and PD-1) were identified in bilateral tumors in the PBS+IgG2 b group; these populations were modestly reduced in the single treatment groups and were largely depleted with the combination treatment (figure 6A). Contralateral tumors from animals treated with rAPMV-4+aCTLA-4 further accumulated activated CD8 T cells, expressing CD11c, Ly6C, CXCR3, and TCF1 (figure 6A), indicating systemic T cell activation. Though not statistically significant due to large variation between animals, supervised analysis confirmed a trend of increased Th/Treg ratios in the treated tumors in all groups and in contralateral tumors in the rAPMV-4+aCTLA-4 group only (figure 6B). CD8/Treg ratios followed a similar trend, though appearing to be primarily driven by aCTLA-4 (online supplemental figure S11a). Furthermore, the dual treatment induced a trend of increasing Th1 and Tfh in the treated tumor, and a modest reduction in Th2 in comparison to rAPMV-4 alone (figure 6C, online supplemental figure S11b). As in the dLNs, the combinatorial treatment counteracted tumor Treg activation measured by surface CTLA-4 and TIM-3, with the greatest reduction observed in contralateral tumors (figure 6D).

Concomitant with Treg suppression and Th1 polarization, the combined treatment induced beneficial remodeling of the CD8 T cell compartment. Despite considerable heterogeneity within each treatment group, treated tumors from animals receiving rAPMV-4+aCTLA-4 had the greatest trend in increasing PD-1+, TCF-1+ Tpex, and all treatments induced a downward trend in dysfunctional CTLA-4+, TIM-3+ CD8 T cells, consistent with the outcomes of the unsupervised analysis. In the contralateral tumor site, the upward trend in PD-1+TCF-1+ Tpex was driven by aCTLA-4, while only the combination therapy resulted in reduction of CTLA-4+TIM-3+ CD8 T cells in the majority of the animals in the group (figure 6E), highlighting the contribution of aCTLA-4 to the relief of systemic immunosuppression in the bilateral tumor model. The dual treatment additionally increased surface CXCR3 on CD8 T cells in the treated tumor and increased CCR6 in the contralateral tumor (figure 6F). These changes were accompanied by a significant increase in Ki67+and GrB+frequencies of CD8 T cells in treated tumors and in GrB+CD8 T cells in contralateral tumors, most notably in response to the combinatorial treatment (online supplemental figure S11d). These observations are indicative of increased CD8 T cell effector function including proliferation and cytotoxic function. Interestingly, many of the above phenotypes had a somewhat bimodal distribution, similar to the tumor growth distributions observed with reduced dosing (online supplemental figure S8c,d). Pearson correlation analysis highlighted CD4 frequency and expression of suppressive T cell markers as the strongest positive correlates with treated tumor mass, while TCF-1 and Tbet MFI on CD4 and CD8 fractions were the strongest negative correlates with treated tumor mass (figure 6G). Critically, rAPMV-4 treatment with or without aCTLA-4 induced an upward trend in tumor-specific T cell responses, confirmed by multiple functional readouts (online supplemental figures S11f–i and S12a,b). Interestingly, treatment with rAPMV-4 (with or without aCTLA-4) induced a more consistent CD8 response to a peptide in the HN of the virus, while the magnitude of response to the tumor-specific AH-1 peptide was more heterogeneous (online supplemental figure S11e). This observation is consistent with the known tendency of tumors to elicit more heterogeneous antigen-specific T cell responses to a broader antigen landscape, while virus-specific responses tend to converge on a more limited repertoire of antigens.^37 38^

### rAPMV-4 induces innate immune remodeling in dLNs and tumors.

While the most striking immune remodeling occurred in the adaptive compartment, we also observed broad changes in innate immune cells in response to single and combinatorial treatment. rAPMV-4 monotherapy treatment increased neutrophil counts and combination therapy boosted NK cell, B cell, monocyte, and neutrophil counts in treated (but not contralateral) dLNs (figure 7A). Analysis of DC subsets revealed the most significant accumulation primarily of cDC2 in response to rAPMV-4 single and combination regimens in treated and contralateral dLNs, and specifically resident cDC2 in contralateral dLNs (figure 7B). DCs (especially migratory DCs) in the treated LNs trended toward more activated states with single and combinatorial therapy compared with PBS/isotype controls (figure 7C), though only the combinatorial treatment regimen demonstrated statistically significant changes.

Similar to the dLNs, treated tumors demonstrated an upward trend in monocyte/macrophage and neutrophil frequencies in response to both single and combinatorial treatments, though only the former population reached statistical significance (figure 7D). While overall DC frequency was not altered in response to therapy, single and combination treatment boosted the proportion of cDC2s (figure 7E).

## Discussion

This study developed a rational combinatorial approach for the treatment of CRC leveraging rAPMV-4, a virotherapy with antitumor efficacy in multiple syngeneic murine CRC models, with low-dose CTLA-4 blockade. This study primarily focused on the MSS CT26 model, selected for its established susceptibility to rAPMV-4 virotherapy and shared features with human CRC subtypes (figure 3G). We demonstrated that in situ vaccination with rAPMV-4 is highly efficacious in single and bilateral tumor models, with the monotherapy inducing complete remissions in treated and contralateral tumor sites. In contrast, bilateral tumor studies performed with NDV, a closely related clinical candidate virotherapy, have demonstrated abscopal effect only in combination with checkpoint inhibitors or payloads.^14 39^

This difference in efficacy may be partially explained by the induction of type I IFN and adaptive immune responses after just a single dose of rAPMV-4. Similar studies using NDV in murine melanoma models have also demonstrated interferon induction; however, this was only reported after three repeated doses.^40^ rAPMV-4 treated animals also had increased levels of CXCL9 and 10 in the serum, both of which are key T cell chemoattractants linked to improved T cell responses and tumor control.^41 42^ While CXCL9 and 10 can be produced in response to IFN by T cells, monocytes, neutrophils, endothelial cells, and some cancers, the primary source of the chemokines in our system remains to be determined.

In addition to the above T cell-specific signatures, GSEA of treated tumors also revealed upregulation of gene sets predictive of response to ICB (online supplemental figure S1b) by a machine learning framework trained on an extensive dataset of murine tumors.^43^ In fact, the predictive gene set most enriched in rAPMV-4 treated tumors was previously associated with the best response to ICB and the most downregulated gene set was associated with the worst response. Further comparison of pathways enriched in PBS control CT26 tumors versus healthy murine colon from ENCODE demonstrated enrichment of metagenes predictive of resistance to ICB, highlighting the inherently immunosuppressive nature of the CT26 tumors (online supplemental figure S1c).

Notwithstanding the potent immunostimulatory effects of rAPMV-4 in situ vaccination in the present model, it has been well established that the use of virotherapeutics as single agents generally is insufficient to induce long-term control of tumor growth and complete responses in the clinic.^44–46^ Informed by our systems approach to characterizing host responses to rAPMV-4, we aimed to both boost existing responses and target mechanisms of resistance with a rationally-designed combinatorial regimen. Given the enriched ICB-response metagenes in rAPMV-4 treated tumors (online supplemental figure S1b,e,f), as well as the heightened similarity with human CMS1 tumors induced by treatment (figure 3G), we postulated that this virotherapy could be combined successfully with ICB. Despite the enrichment of these positive prognostic signatures, tumors treated with the vector retained a persistent Treg transcriptional signature (figure 3C), which is critical considering Tregs have been linked to immunotherapy resistance.^47^ Due to these factors, we chose to combine rAPMV-4 with low-dose aCTLA-4, given its ability to deplete^36^ Tregs and impair their immunosuppressive activity while boosting effector T cell function.

As predicted by our cellular and transcriptomic signatures, the addition of aCTLA-4 to the virotherapy increased complete responses in treated and contralateral tumors, and induced T cell expansion in tumors and dLNs that surpassed that of either monotherapy. NDV has also been used in conjunction with aCTLA-4 (through both payload incorporation or combination treatment),^14^ boosting its efficacy in animal studies. Our findings, however, highlight rAPMV-4 as a more efficacious vector in this tumor model with more potential for synergy with aCTLA-4 than NDV (online supplemental figure S8b–d). Other, non-paramyxovirus virotherapies have been explored in combination with aCTLA-4, with one of the most notable being T-VEC, a herpesvirus-1 expressing GM-CSF (a cytokine that recruits and activates antigen-presenting cells). A phase II clinical trial evaluating the efficacy of the combination of T-VEC and ipilimumab revealed a significant advantage of the combinatorial approach for melanoma,^48^ while, interestingly, the combination of T-VEC with pembrolizumab, a PD-1 inhibitor did not recapitulate this benefit.^49^ These divergent outcomes indicate that not all T cell-targeted therapies are equal in combination with virotherapies. While the above studies did not include any biomarker analysis that could explain these results, it is possible that the anti-Treg functionality of CTLA-4 blocking antibodies may be better at counteracting homeostatic immunosuppressive responses to virotherapy in these settings. While this feature may depend on the particular biology of a given vector and tumor model, several reports have highlighted heightened the potential role of Tregs in resistance to oncolytic viruses in vivo.^50–53^

Underlying the antitumor efficacy of the rAPMV-4+aCTLA-4 combinatorial therapy, we detected widespread changes in T cell phenotypes in tumors and dLNs. The combination induced preferential expansion of canonically antitumoral T cell fractions, including CD8 T cells, Th1, and Tfh, to a greater degree than either of the monotherapies. While CD8 T cells act as the primary antitumor cytotoxic cell subtype, Th1 and Tfh provide important support for the antitumor immune response. Th1 plays a key role in supporting CD8 effector responses by participating in antigen presentation triads along with DCs and providing activating cytokines, while Tfh are key mediators of antibody generation. Generally, the effect was the most pronounced in the treated tissues of animals receiving the combinatorial treatment, while the systemic aCTLA-4 drove the antitumoral shift in T cell frequencies in the contralateral tissues.

Despite the increase in effector and Th frequencies in response to rAPMV-4, the monotherapy failed to significantly reduce Tregs in dLNs. Further, a subset of animals suffered from an increase in highly suppressive Tregs expressing CTLA-4, TIM-3 (canonical markers of effector T cell exhaustion, which also contribute to the suppressive function of Tregs when expressed on their surface), or both in dLNs (figure 5F), indicating a potential mechanism of resistance to the virotherapy. Tumors treated with rAPMV-4 alone retained CTLA-4 and TIM-3 surface levels on Tregs that were comparable to those in PBS controls (figure 6D). Considering that the dLN population with the strongest correlation with tumor mass was Tregs (figure 5H), this cell type and its activation status could be a major contributing factor to resistance to rAPMV-4.

Indeed, Tregs are well known for their multimodal suppressive functions on cytotoxic CD8 T cells, Th cells, B cells, and DCs such as competition for IL-2, release of suppressive cytokines (ie, TGF-β and IL-10), and binding of inhibitory ligands such as CTLA-4 and LAG-3 to DCs.^54^ Multiple studies have reported a correlation between Treg infiltration in colorectal tumors and worse clinical outcomes, including liver metastasis.^55 56^ Murine studies have underscored the importance of Tregs in colorectal cancer progression,^57^ with TIM-3+Tregs in particular shown to drive T cell inhibition through mechanisms such as TGF-β and IL-10 secretion.

In our model, the combination of aCTLA-4 with rAPMV-4 reversed immunosuppressive Treg phenotypes in dLNs and tumors. This effect was most pronounced in contralateral tumors, suggesting a critical synergy between the two treatments in overcoming resistance at distal tumor sites. This reduction in Treg functionality was also associated with heightened indicators of Th and T effector functionality and enhanced DC activation. Specifically, the combination treatment most effectively reduced markers of exhaustion on CD8 T cells and promoted their activation and migration. CXCR3 was significantly upregulated on CD8 T cells in both dLNs and treated tumors, and CCR6 in the contralateral dLNs and tumors. CXCR3 has a critical role in mediating CD8 T cell migration to sites of inflammation via interaction with its ligands, CXCL9 and CXCL10,^58^ both of which were upregulated in the serum of animals treated with rAPMV-4 (figure 3F). CCR6+CD8 T cells have been described as consistent with early effector memory cells and respond to CCL20, suggesting their ability to migrate to the colon.^59^ In conjunction with improved T cell migratory capacity, combination treatment also induced an upward trend in Tpex frequency in tumors (figure 6E), which are associated with response to immunotherapy given their ability to respond to antigen and undergo self-renewal.

Several studies have reported a mechanism for CTLA-4 mediated T cell suppression through an alternatively spliced soluble CTLA-4 variant^60–62^ that can also be targeted with CTLA-4-blocking antibodies. While we were able to detect low levels of CTLA-4 in the serum of CT26 tumor-bearing mice, there was no difference between rAPMV-4 and rAPMV-4+aCTLA-4 treatment (online supplemental figure S8f), suggesting that our therapy is predominantly targeting membrane-bound CTLA-4.

Given the ability of rAPMV-4 to induce T cell activation and migration in treated and contralateral sites in combination with low-dose aCTLA-4, we propose this novel in situ vaccination strategy for future development for the treatment of CRC. The most successful immunotherapy approaches for CRC employ a combination of PD-1 and CTLA-4 blockade, which, while inducing OR in 69% of patients in a recent trial,^63^ causes frequent adverse effects. Additionally, ICB is most effective in patients with MSI tumors, with MSS tumors generally unresponsive to therapy.^64^ Here, we provide evidence of single and double agent immunotherapy efficacy in a murine MSS model.

To interrogate the translational relevance of our model, we leveraged a cohort of treatment-naive human CRC primary tumors. Transcriptionally, untreated CT26 tumors were most similar to human CMS4 tumors, generally the most immunosuppressive and treatment-resistant subtype. rAPMV-4 treatment shifted murine signatures toward CMS1 (figure 3G), which is characterized by high immune infiltration and considered to have the best response to ICB out of all the CMS types, underscoring the potent immunostimulatory properties of the virotherapy.

Given this shift and the robust T cell remodeling induced by rAPMV-4 in our murine model, we aimed to evaluate the utility of a temporally stable virotherapy T cell response signature in predicting CMS1 patient survival outcomes. Out of the input gene list, SOCS1, RUNX3, and GADD45G had the most significant negative risk influence in patients, which was confirmed by multivariate and univariate survival analysis (figure 3H, online supplemental figure S5a–c). Of note, our T cell response signature was not useful in stratifying CMS4 patients by risk, likely due to the more immunosuppressive TME of this subtype that antagonizes effector T cells and promotes metastasis.

The above three genes have diverse biological functions that are microenvironment and cell type dependent, broadly including T cell immunity and tumor suppression. As SOCS1 is a negative regulator of the JAK/STAT pathway and inhibits IL-6 and IFNγ signaling, it may be critical in preventing chronic inflammation, which impairs antitumor immunity and is a common feature of CRC.^65^ While there are some conflicting studies on the effects of SOCS1 in the establishment of antitumor immunity, it is reported to be repressed in more aggressive CRC tumors,^66^ and has roles in generating noncanonical class 1 peptides and suppressing IFNγ-induced PD-L1^65^ expression to enhance tumor-specific T cell priming and prevent exhaustion.

RUNX3, in addition to its canonical role in T cell development, can also promote expression of cytotoxic molecules in CD8 T cells and can promote memory T cell differentiation.^67^ Elevated frequency of RUNX3+CD8+ T cells in tumors has been shown to associate with highly significant improvements in survival.^68^ When expressed by cancer cells, it is a tumor suppressor and represses metastasis in CRC through inhibition of hedgehog signaling,^69^ highlighting its multimodal anticancer functions. Unlike the previous two genes, GADD45G has been primarily described in responses to DNA damage and cell cycle arrest, though it was also shown to promote Th1 responses when expressed in DCs.^70^

Considering how the three genes may affect the tumor and its immune microenvironment, we can infer that repression of chronic inflammation occurs simultaneously with enhancement of CD8 and Th1 function in CMS1 patients with more favorable survival outcomes. Future studies will benefit from further investigation of the prognostic value of this gene set in other patient cohorts, or its potential utility in predicting immunotherapy response. Induction of these signatures by rAPMV-4 in human samples, as well as their mechanistic contribution to the virotherapy, will need to be validated in the future using human organoids or patient-derived explants. Nonetheless, the temporally conserved induction of these critical genes by rAPMV-4 in murine tumors, and their robust associations with patient survival outcomes suggest much promise for this vector in priming resistant tumors for improved immunotherapy responses.

While CT26 recapitulates key transcriptional and immune features of the aggressive, treatment-refractory CMS4 subtype of human CRC, colorectal cancer is a heterogeneous disease encompassing tumors with distinct genetic backgrounds and immunosuppressive TMEs. Future studies will extend this systems-level profiling approach to additional syngeneic CRC models spanning this heterogeneity, to further define the breadth of tumor contexts in which rAPMV-4-based combination virotherapy is effective.

All in all, we found that rAPMV-4 monotherapy rapidly induced signatures of antigen presentation and T cell activation in vivo, including transcriptional alterations predictive of response to ICB in preclinical models. A combinatorial treatment approach with CTLA-4 blockade, selected for augmentation of observed T cell responses to the virotherapy as well as emerging pathways of resistance, reversed Treg activation and effector T cell dysfunction and significantly boosted complete responses in bilateral tumors. Importantly, this work represents a strong foundation for rAPMV-4, either alone or in combination, for further clinical development of a more efficacious and safer treatment regimen for CRC patient populations.

### Statement of significance

Oncolytic virotherapy is a promising strategy for enhancing antitumor immunity by inducing both innate antiviral responses and in situ T cell priming. Here, we present rAPMV-4, a novel avian avulavirus platform with broad and potent immunostimulatory capabilities that shows promise in sensitizing wider colorectal cancer patient populations to immunotherapy.

## Supplementary material

10.1136/jitc-2026-015859 online supplemental file 1

10.1136/jitc-2026-015859 online supplemental table 1

10.1136/jitc-2026-015859 online supplemental table 2

10.1136/jitc-2026-015859 online supplemental table 3

## Data Availability

Data are available on reasonable request. All data relevant to the study are included in the article or uploaded as supplementary information.
