# Supplementary material for: In situ APMV-4 vaccination primes systemic response to subtherapeutic dosing of aCTLA-4 in colorectal carcinoma
Source: J Immunother Cancer. 2026 Sep 28;14(9):e015859. doi: 10.1136/jitc-2026-015859 (PMC13629906; doi:10.1136/jitc-2026-015859)
Supplement: Supplementary file 1 [file jitc-14-9-s001.pdf]

**Supplementary Figure S1**

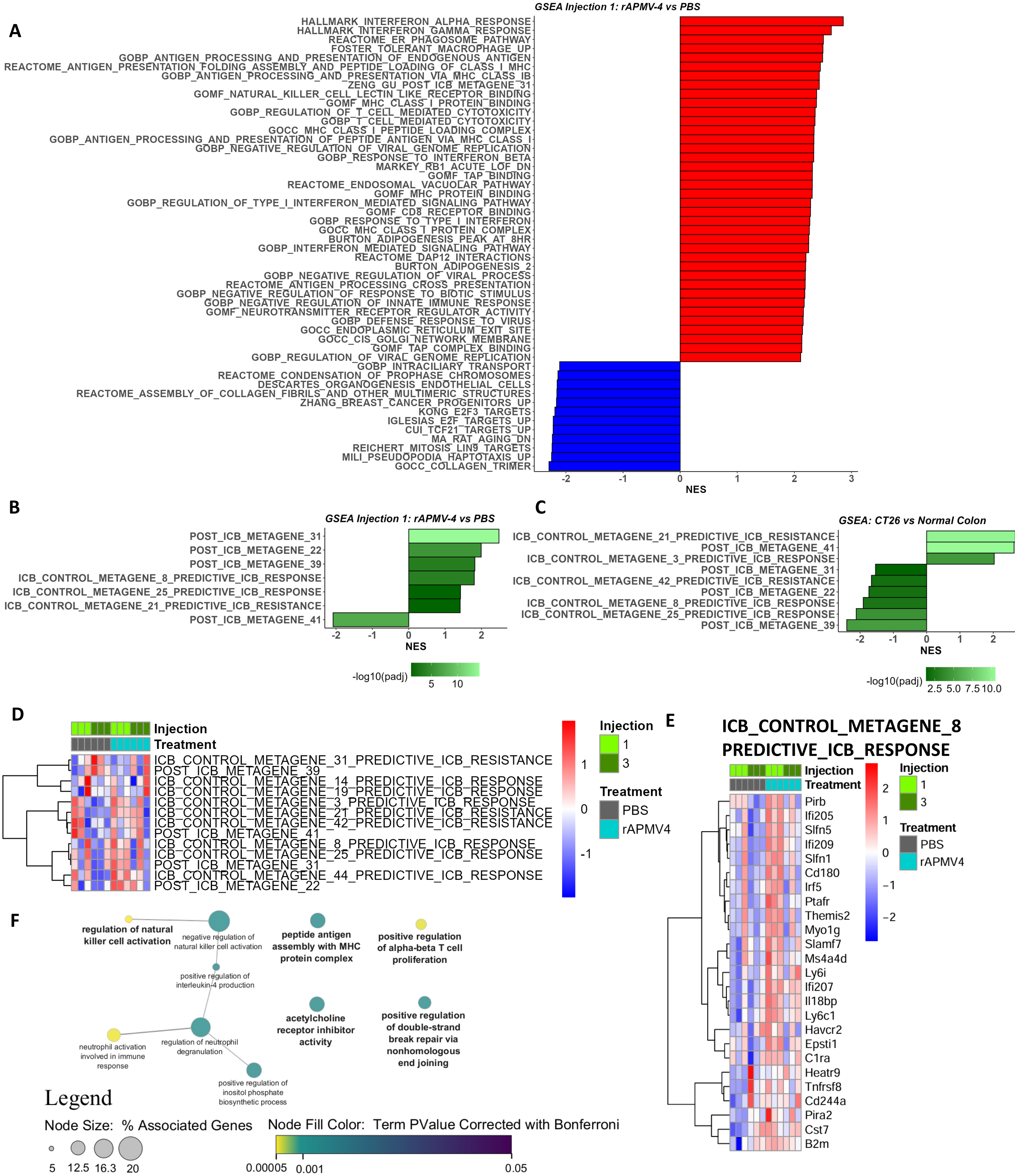


**Figure S1: rAPMV-4 monotherapy promotes rapid induction of antiviral inflammatory programs and ICB response modules**. **A,** Barplot representing top 50 pathways from GSEA of Injection 1 rAPMV-4 vs PBS treated tumors. **B,** Barplot representing ICB response modules (metagenes) from GSEA of Injection 1 rAPMV-4 vs PBS treated tumors. **C,** Barplot representing ICB response modules (metagenes) from GSEA of CT26 PBS treated tumors vs murine normal colon from the ENCODE database. **D,** Heat map depicting GSVA for individual tumors filtered on ICB response/resistance metagenes. **E,** Heat map of relative expression of top 25 differentially expressed leading edge genes for Metagene 8 predictive of ICB response. **F,** ClueGo pathway network analysis for leading edge genes for Metagene 8.

**Supplementary Figure S2**

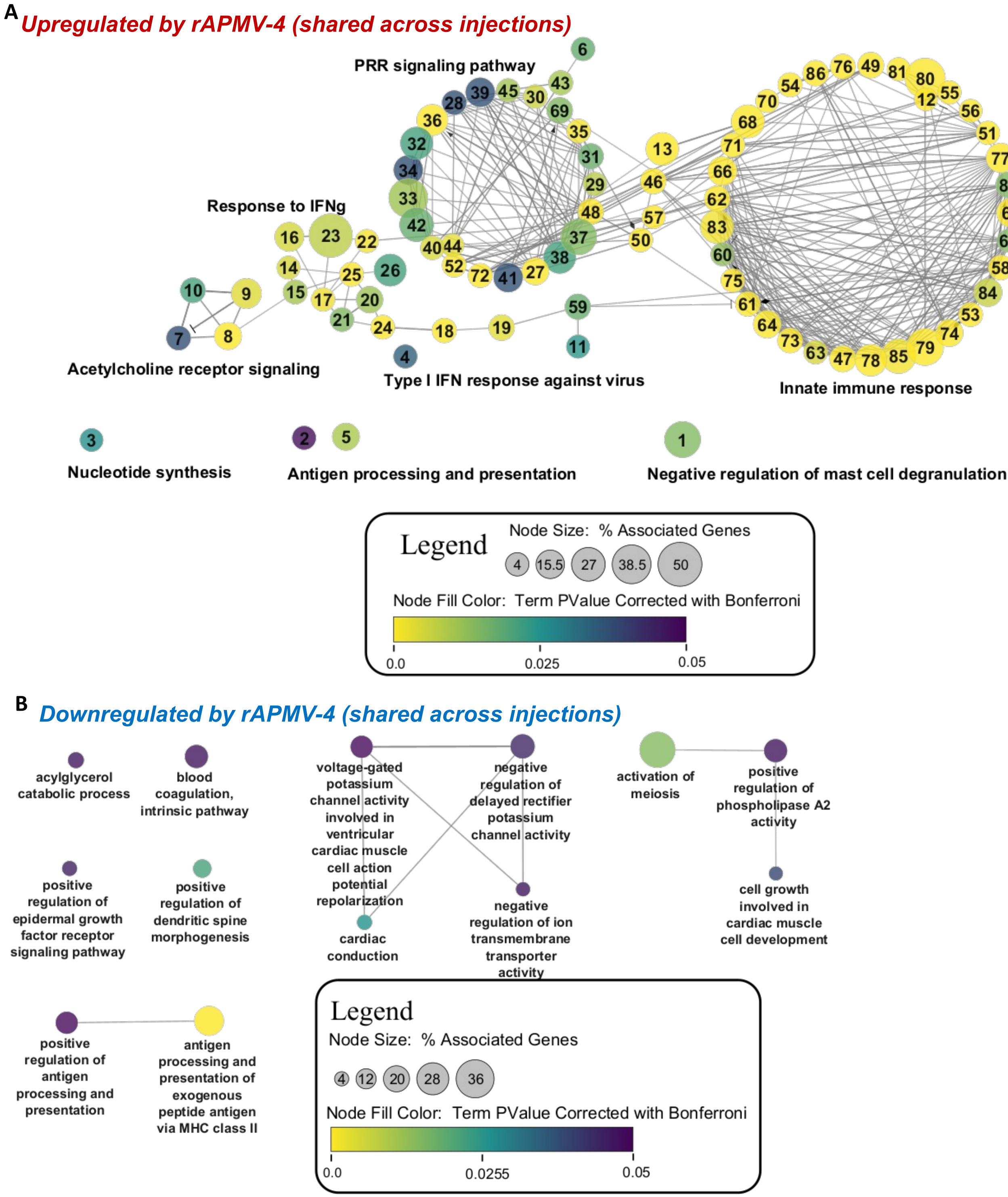


**Figure S2: rAPMV-4 treatment induces sustained antiviral response, antigen presentation and apoptotic pathways. A,** ClueGo pathway network analysis for genes upregulated by rAPMV-4 at both injection timepoints, filtered from top 200 upregulated genes from both timepoints. **B,** ClueGo pathway network analysis for genes downregulated by rAPMV-4 at both injection timepoints, filtered from top 200 downregulated genes from both timepoints.

## Supplementary figure S3

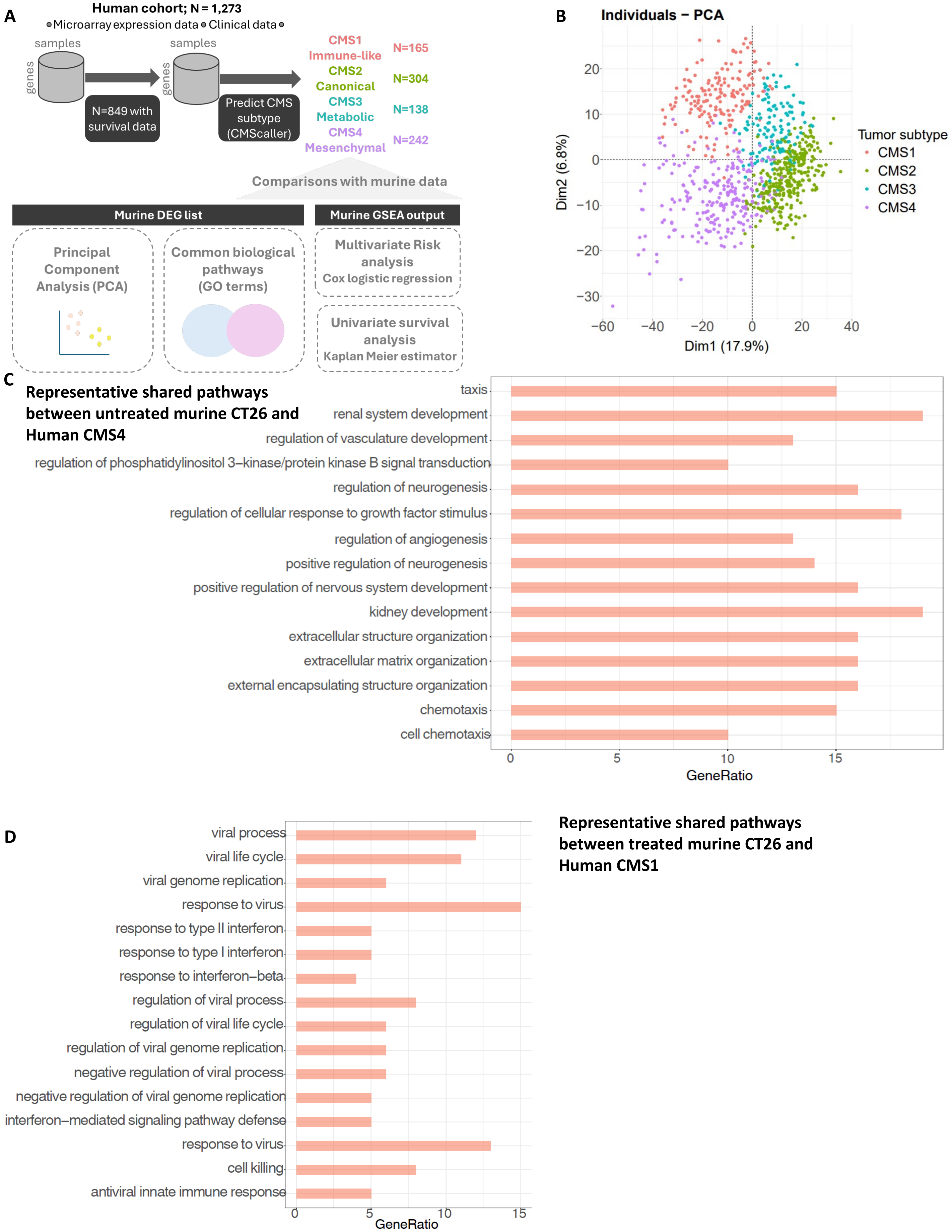


**Figure S3**: **CMS classification reliably groups CRC patients and shares biological pathways with murine CT26 tumors. A,** Human transcriptomic analysis workflow schematic. **B,** PCA plot of human CRC primary tumors using DEG list from rAPMV-4 vs PBS treated tumors. **C,** Barplot with representative GO pathways shared between murine untreated (PBS control) tumors and human CMS4 tumors. **D,** Barplot with representative GO pathways shared between murine treated (rAPMV-4) tumors and human CMS1 tumors.

## Supplementary Figure S4

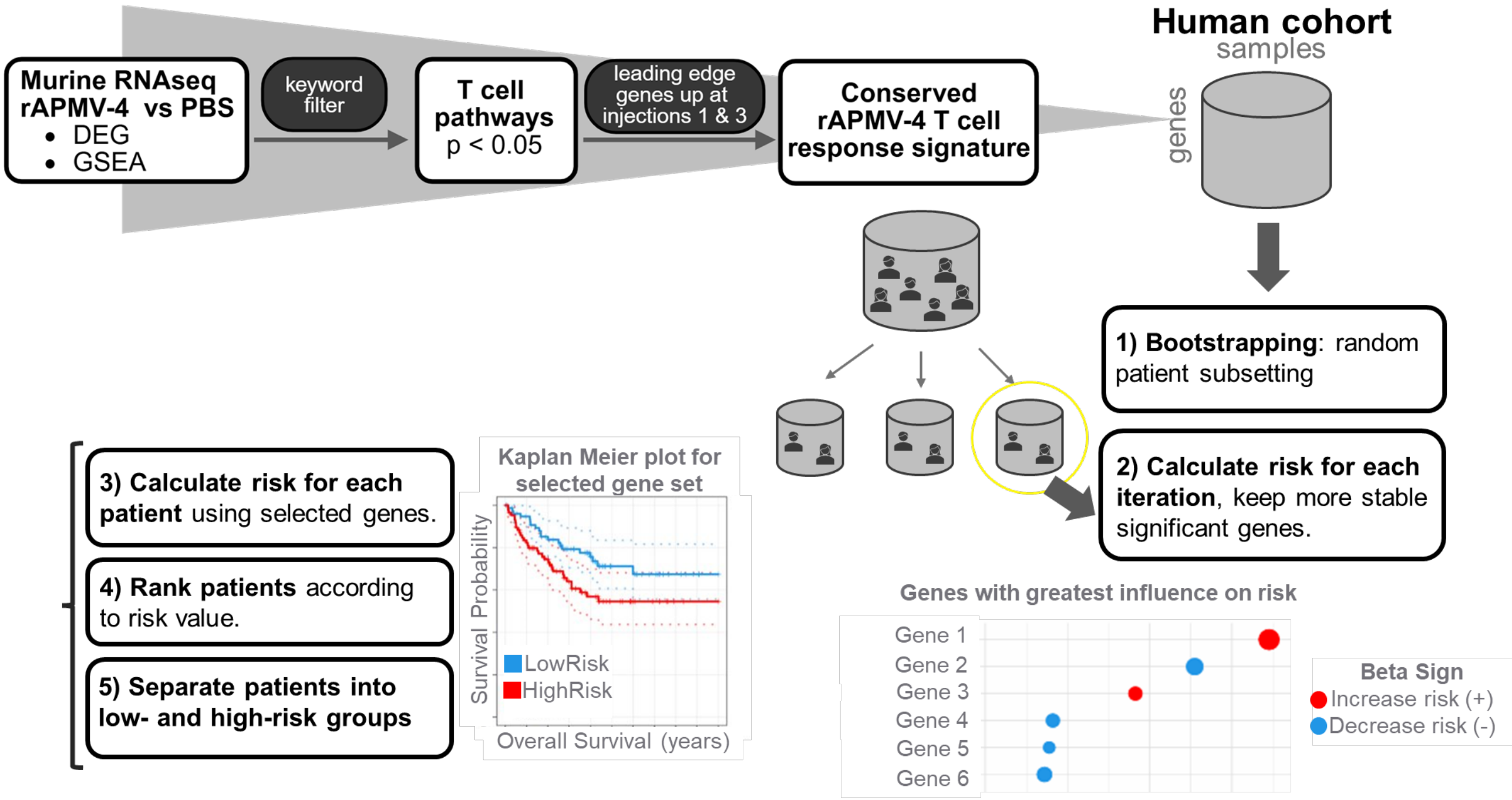


**Figure S4: Risk analysis of virotherapy T cell response gene set in human CRC cohort**. Schematic for gene set selection and risk analysis in human cohort.

## Supplementary Figure 5

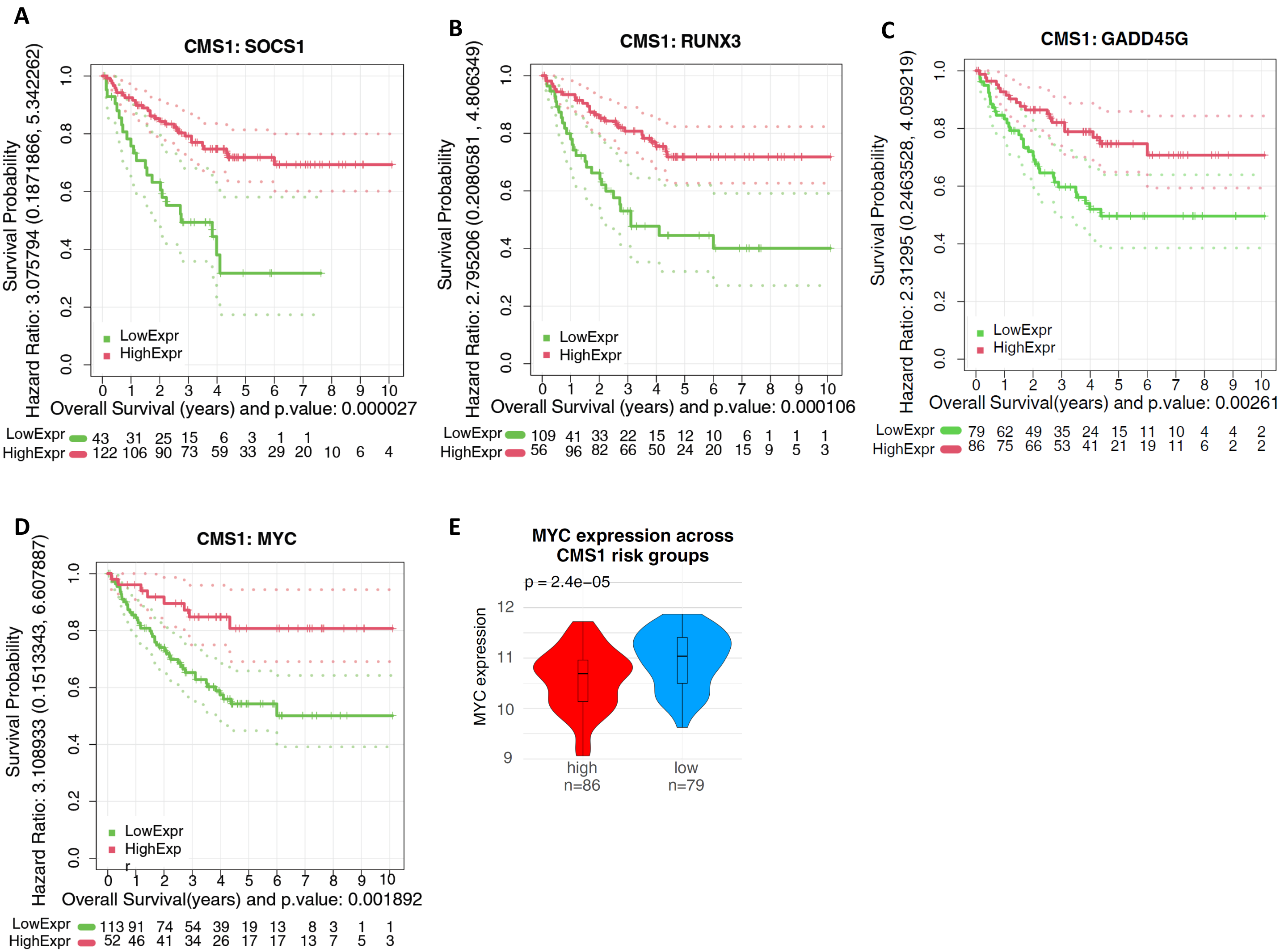


**Figure S5: Univariate survival analysis for top virotherapy T cell gene set negative risk contributors for CMS1**. **A-D:** Kaplan-Meier plots for univariate survival analysis in CMS1 patient cohort. Survival analysis based on low vs high expression of **A;** SOCS1, **B;** RUNX3, **C;** GADD45G, **D;** MYC expression. **E**, Violin plot for relative expression of MYC, divided by risk group.

**Supplementary Figure S6**

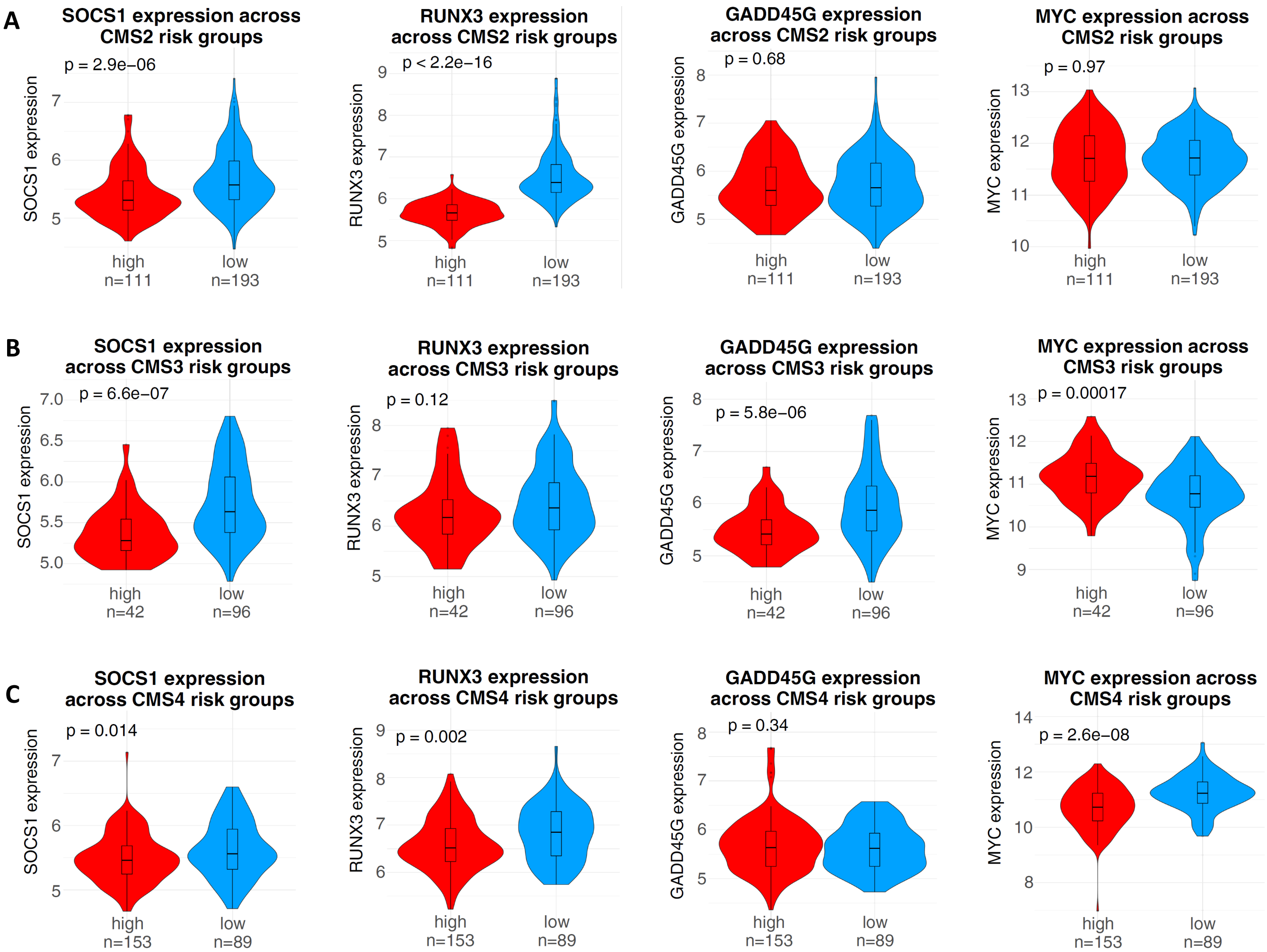


**Figure S6: Select T cell associated gene expression in CMS2, CMS3, and CMS4 risk groups.** Violin plots for relative expression of selected top genes with influence on risk from multivariate risk analysis, divided by CMS and risk group. **A,** Expression across CMS2 risk groups. **B,** Expression across CMS3 risk groups. **C,** Expression across CMS4 risk groups.

## Supplementary Figure S7

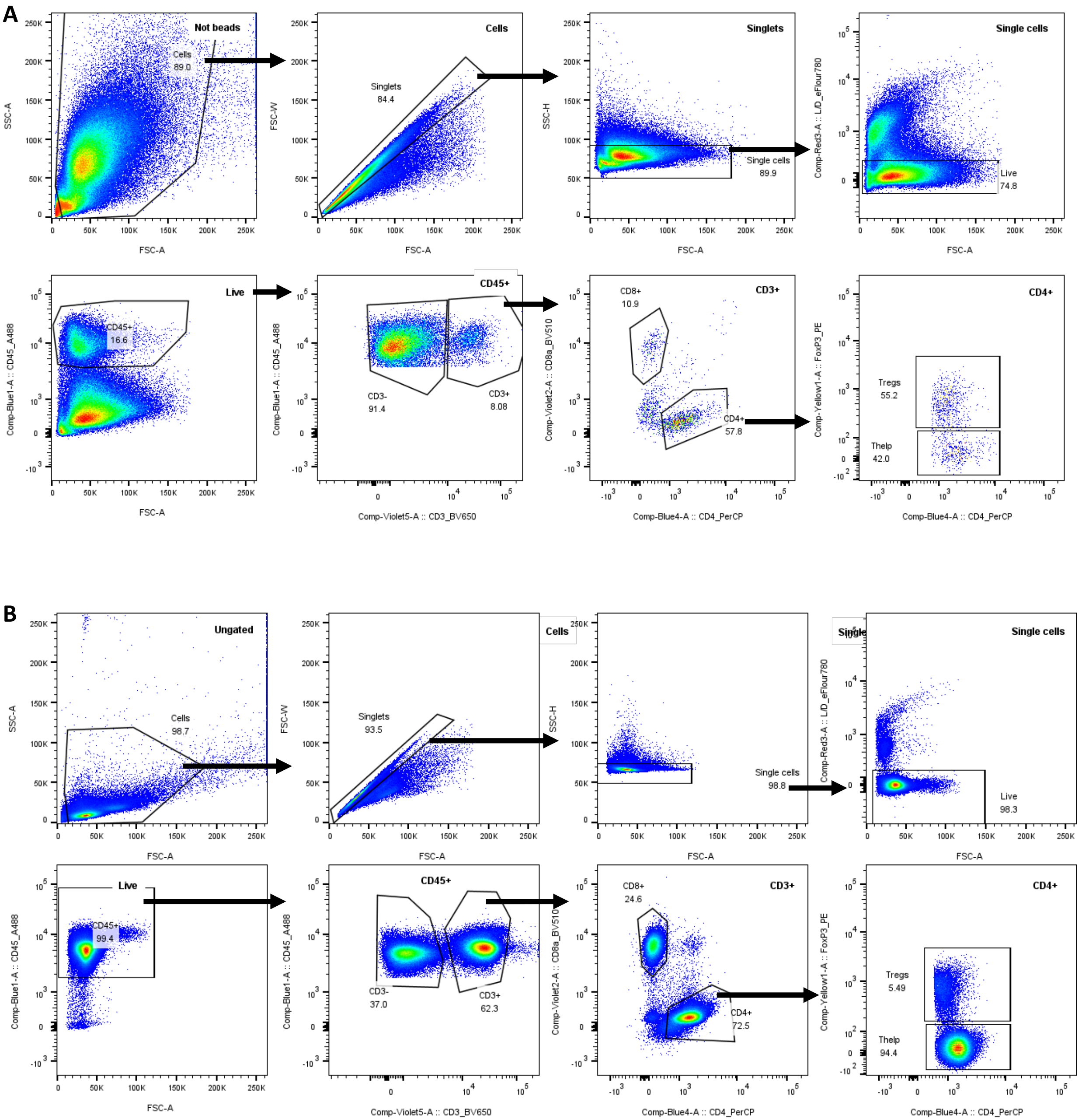


**Figure S7**: Conventional flow cytometry gating strategy. **A,** Tumor gating strategy. **B,** dLN gating strategy.

**Supplementary Figure S8**

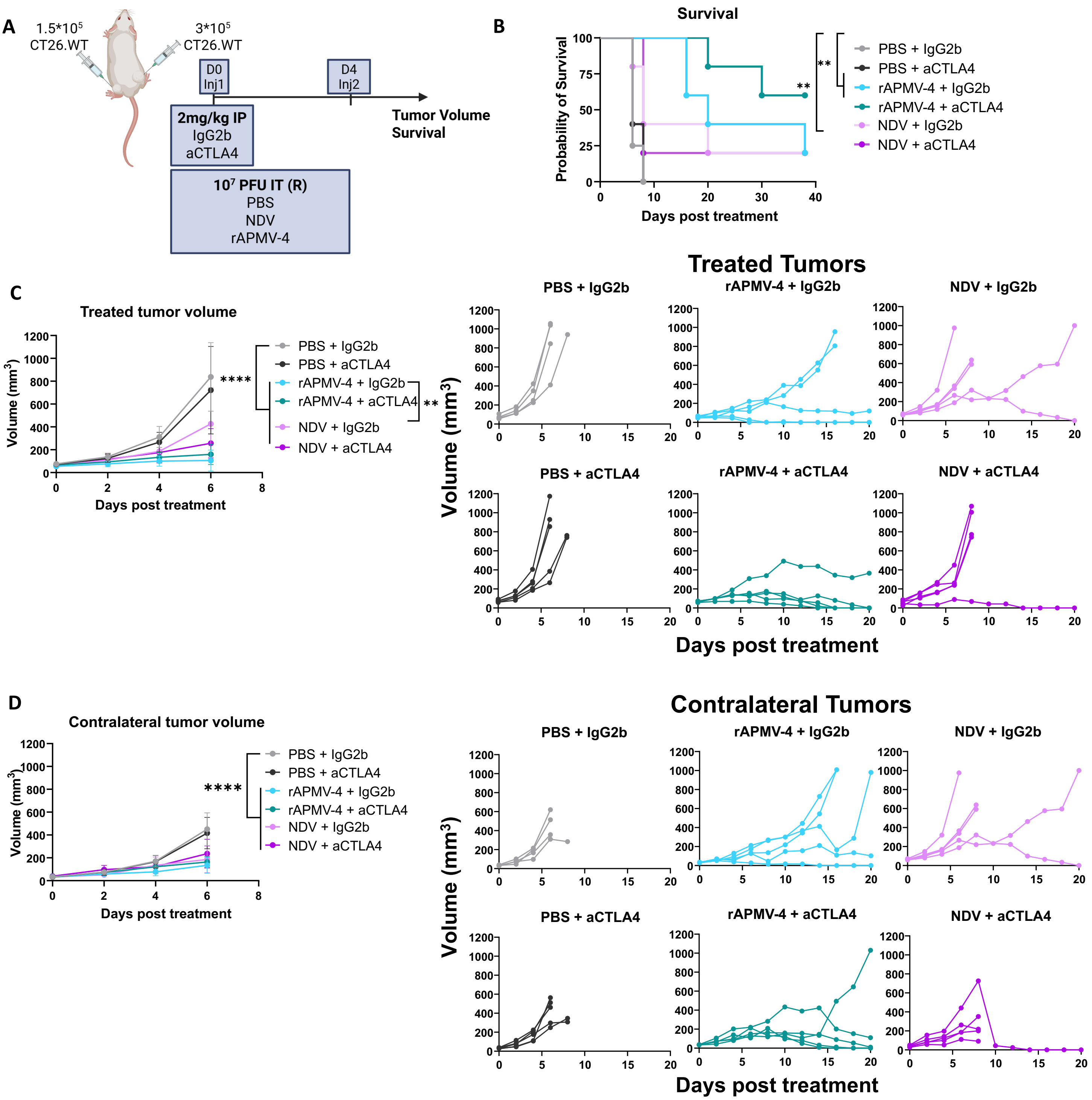


**E**

| Treatment | Treated Tumor CR | Contra. Tumor CR |
|---|---|---|
| PBS + IgG2b | 0/4 | 0/4 |
| PBS + aCTLA-4 | 0/5 | 0/5 |
| rAPMV-4 + IgG2b | 2/5 | 1/5 |
| rAPMV-4 + aCTLA4 | 4/5 | 3/5 |
| NDV + IgG2b | 1/5 | 1/5 |
| NDV + aCTLA4 | 1/5 | 1/5 |

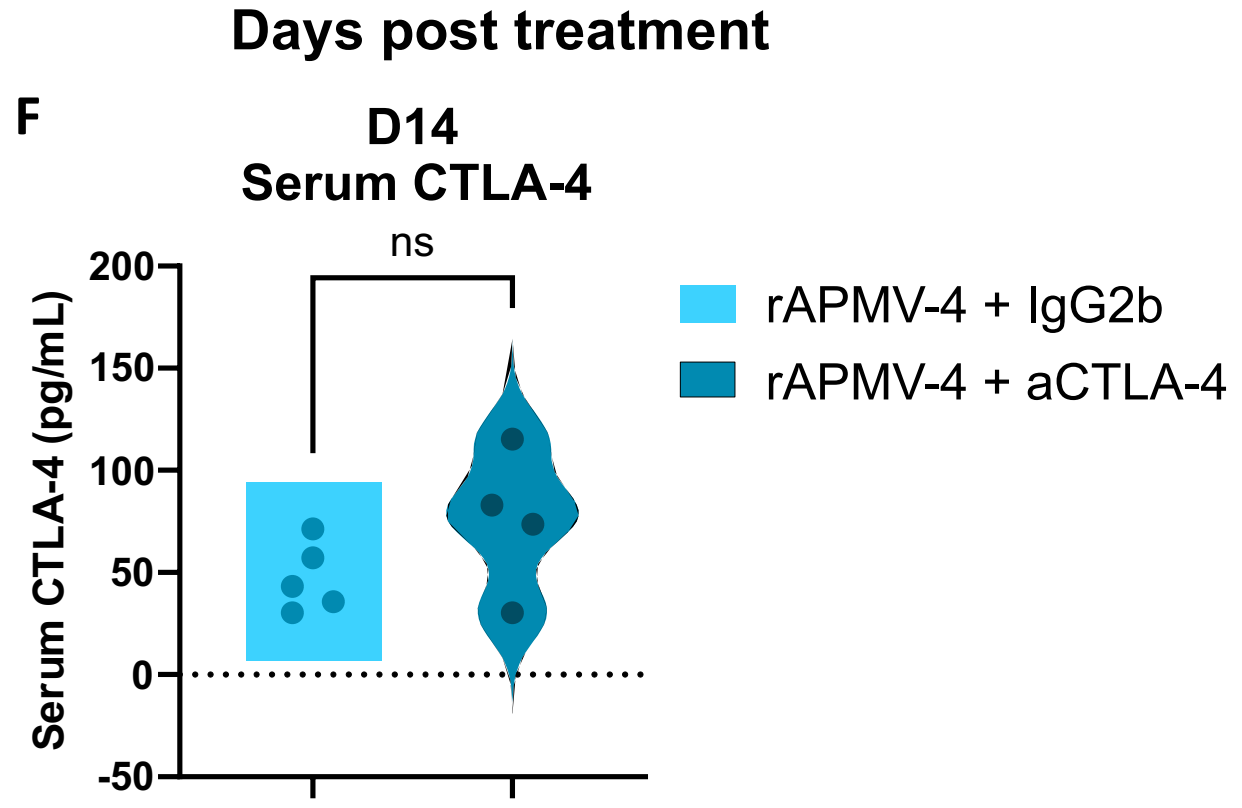


**Figure S8: Low dose aCTLA-4 coadministration with rAPMV-4 boosts antitumor effect of monotherapies and outperforms NDV**. **A,** *In vivo* treatment schematic for the bilateral tumor model in **B-F**; N = 4-5 animals per group. **B,** Kaplan-Meier survival analysis, with p values calculated using log-rank (Mantel–Cox) statistical analysis. **C-D:** Average (left) and individual (right) tumor volumes +/- SEM, with statistical analysis shown from two-way anova at time of first death. **C,** Treated (injected) tumors and **D,** contralateral (uninjected) tumors. **E,** Total complete remissions of treated and contralateral tumors represented in C-D. **F,** Soluble CTLA-4 detected in serum at D14 post treatment initiation (only groups with sufficient animals for statistical analysis were included).

**Supplementary Figure S9**

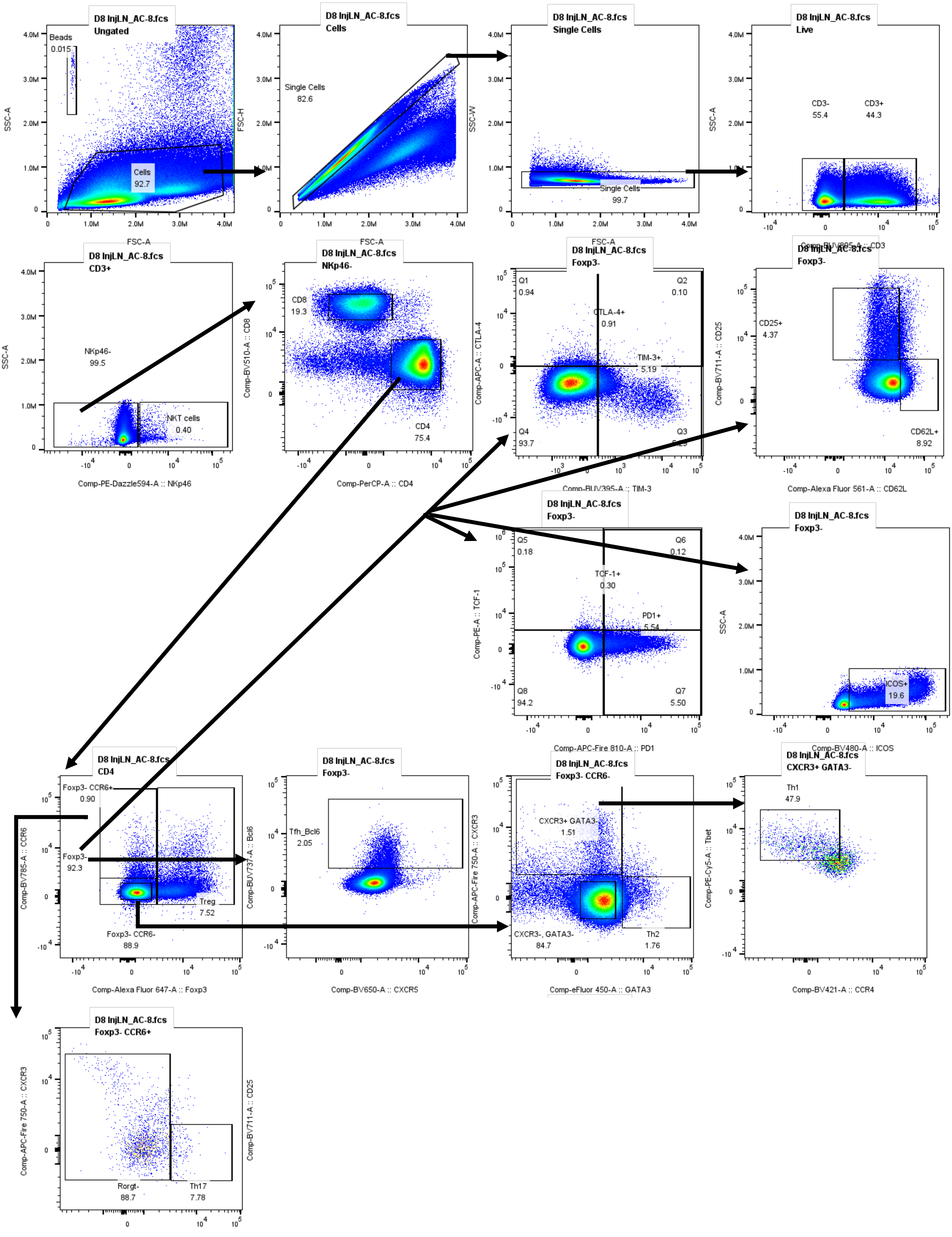


**Figure S9: Spectral flow cytometry gating strategy, CD3+ fraction.**

**Supplementary Figure S10**

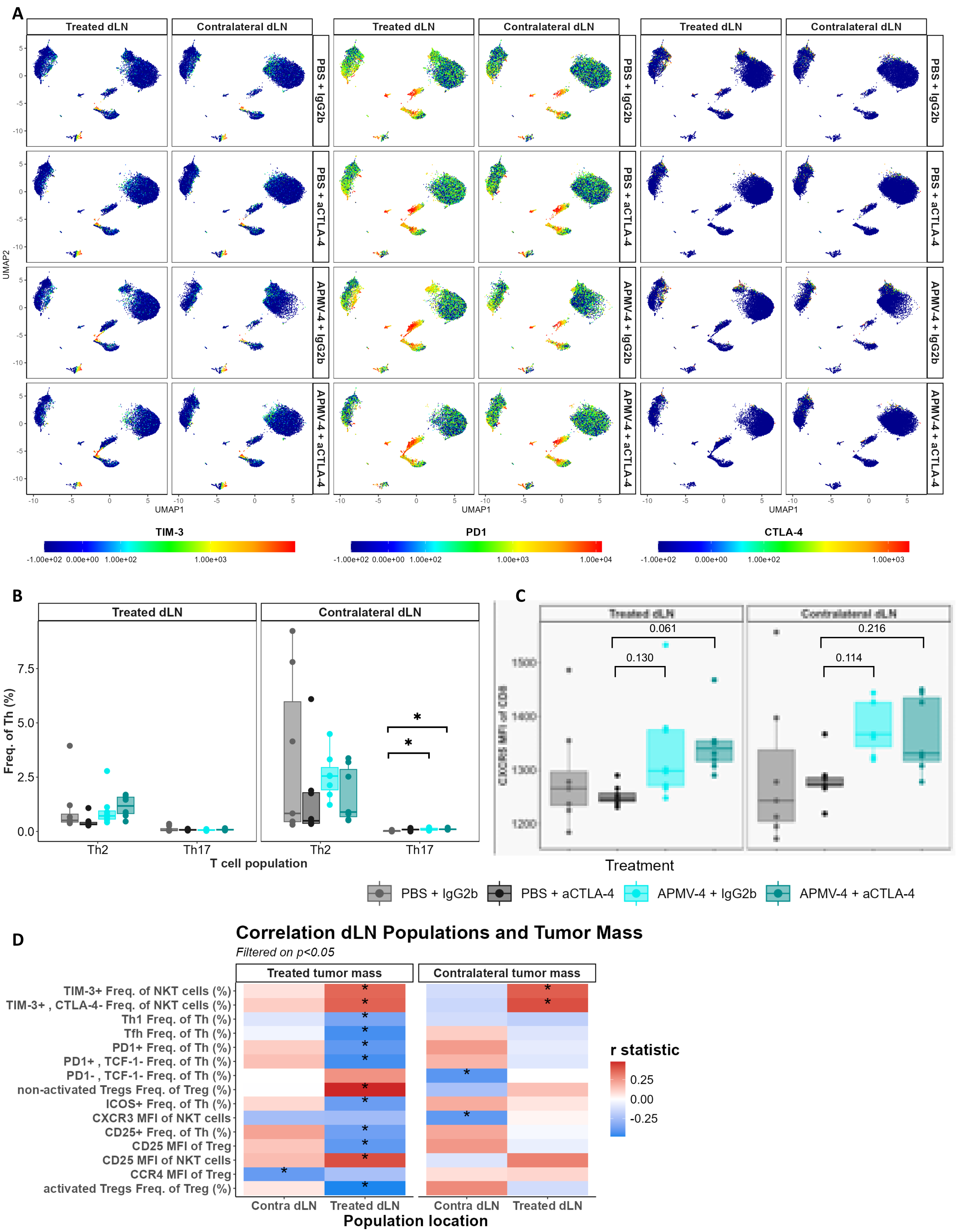


**Figure S10: rAPMV-4 combined with low-dose aCTLA-4 prevents T cell exhaustion in treated and contralateral tdLN. A,** TIM-3 (left), PD1 (middle), and CTLA-4 (right) heatplots visualized on UMAP projections of treated and contralateral dLN from all treatment groups. **B,** Th frequencies in treated and contralateral dLNs identified by supervised analysis. **C,** CXCR5 MFI on CD8 T cells identified by supervised analysis. **D,** Pearson correlation analysis of clusters identified by supervised analysis of treated and contralateral (contra) dLN and treated and contralateral tumor masses, with color representing the r statistic. Only clusters that yielded a significant p value for at least one tissue site are shown here.

## Supplementary Figure S11

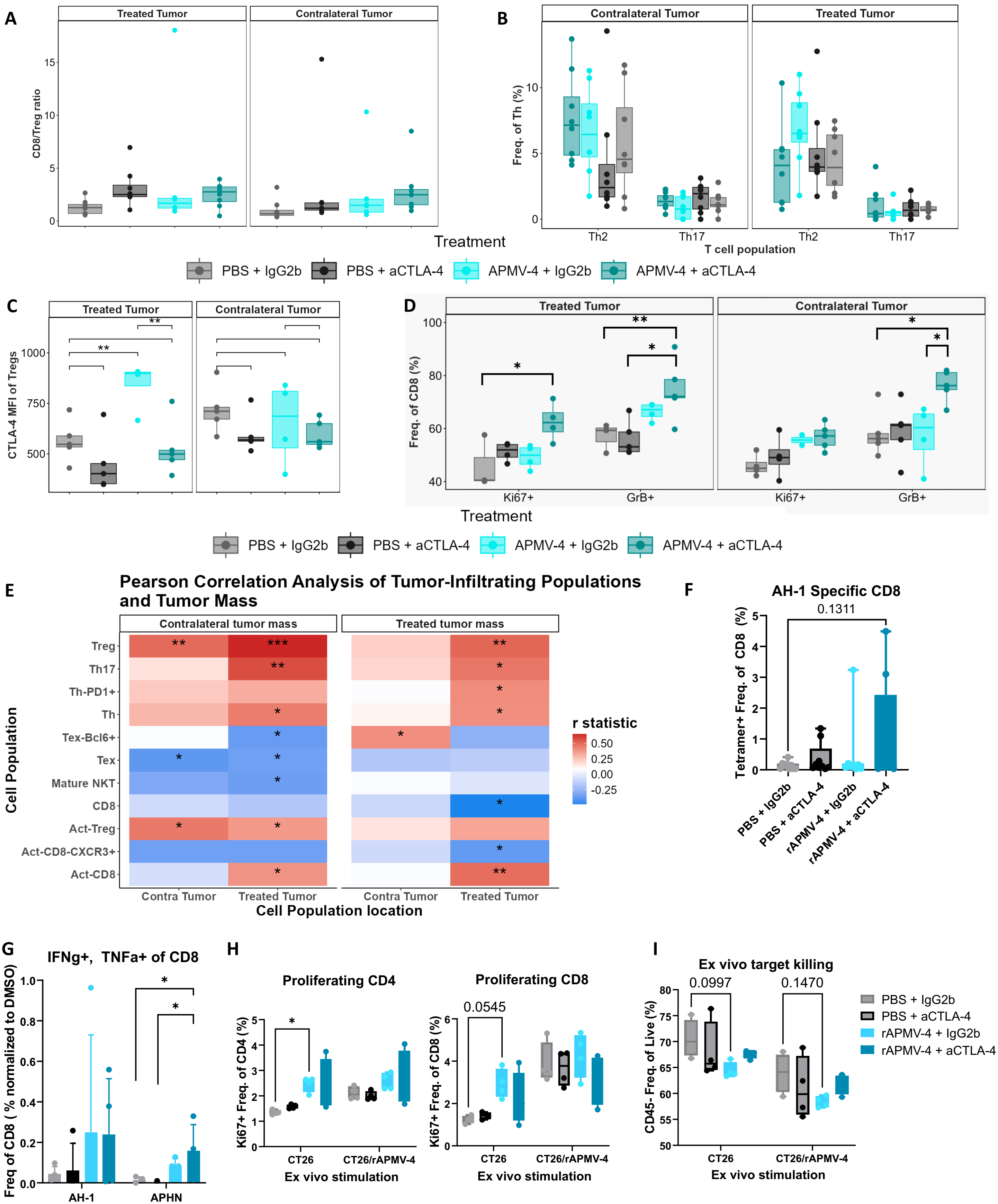

**Figure S11: rAPMV-4 combined with low-dose aCTLA-4 induces T cell recruitment into treated and contralateral tumor. A,** CD8/Treg ratios in treated and contralateral tumors. **B,** Th frequencies. **C,** CTLA-4 MFI on Tregs in treated and contralateral tumors. **D,** Frequencies of Ki67+ and GrB+ CD8 T cells of total CD8 T cells in treated and contralateral tumors. **E,** Pearson correlation analysis of clusters identified by unsupervised analysis of treated and contralateral (contra) tumors and treated and contralateral tumor masses, with color representing the r statistic. Only clusters that yielded a significant p value for at least one tissue site are shown here. **F-I:** Splenocytes from animals treated 3 times with IgG2b/CTLA-4 and PBS/rAPMV-4 as depicted in Fig 5A. **D,** Frequency of AH-1 tetramer+ CD8 T cells (aggregate data from two independent repetitions with n = 4-5 for each repetition). **E,** IFNg+, TNFa+ CD8 T cell frequency after a 6 hr peptide restimulation. **F,** Ki67+ CD4 (left) and CD8 (right) T cells after 24 hr coculture with mock infected or rAPMV-4 infected CT26 cells. **G,** Target cell frequency remaining after 24 hr coculture of splenocytes with mock infected or rAPMV-4 infected CT26 target cells. APHN = peptide derived from HN protein of rAPMV-4 (YYLNGNIYL).

**Supplementary Figure S12**

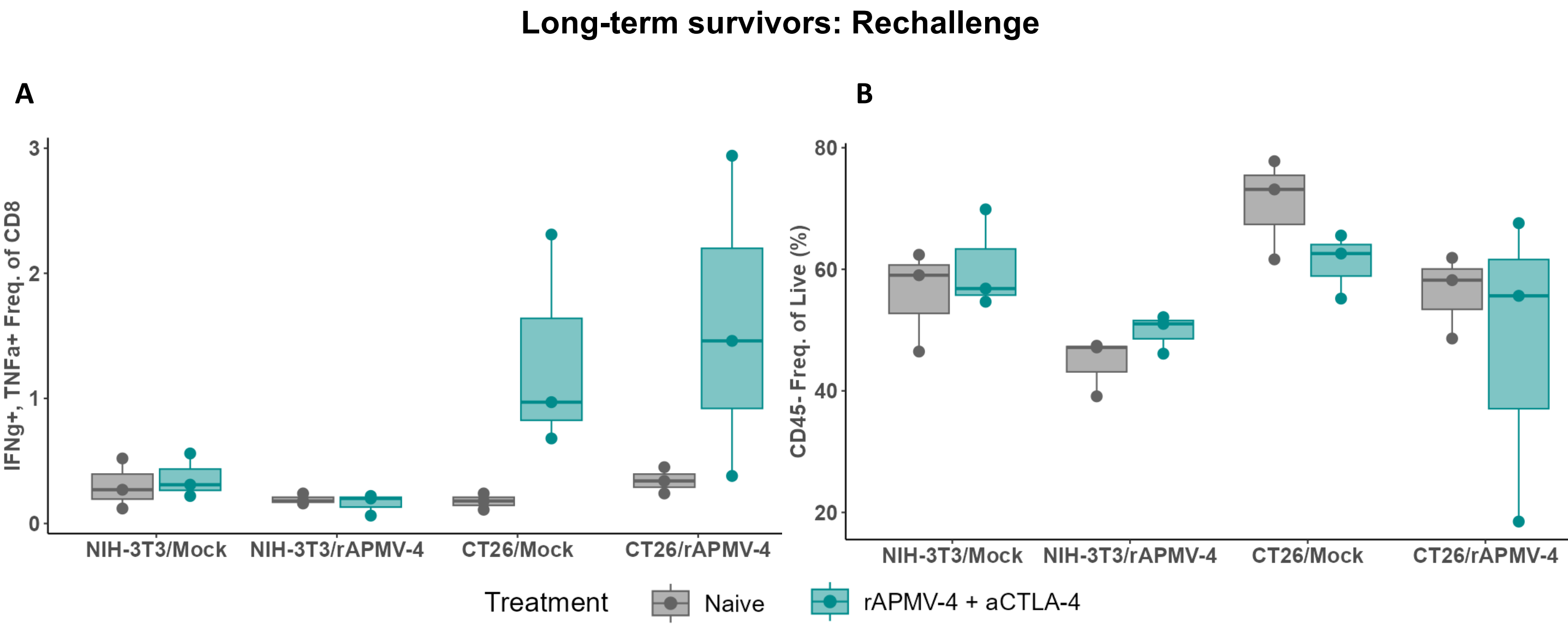


**Figure S12: rAPMV-4 combined with low-dose aCTLA-4 induces tumor-specific T cell memory A,** IFNg+, TNFa+ CD8 T cell frequency and **B,** target cell frequency (right) after a 24 hr coculture of splenocytes (with CT26 or NIH-3T3 fibroblast controls) from complete responders and age-matched naive mice rechallenged with CT26.

## Supplementary Figure S13

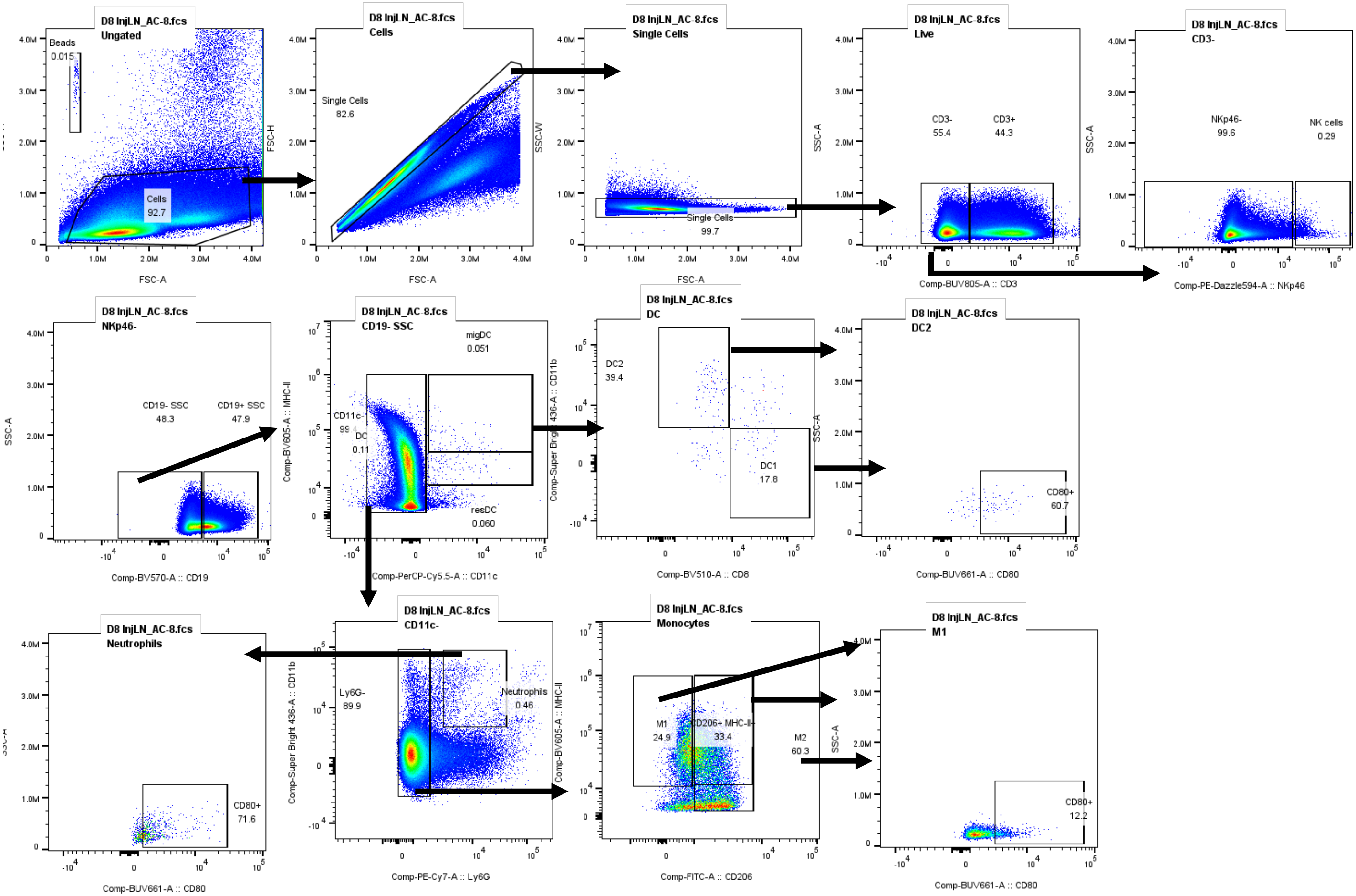


**Figure S13:** Spectral flow cytometry gating strategy, CD3- fraction.

Supplementary Fig S14

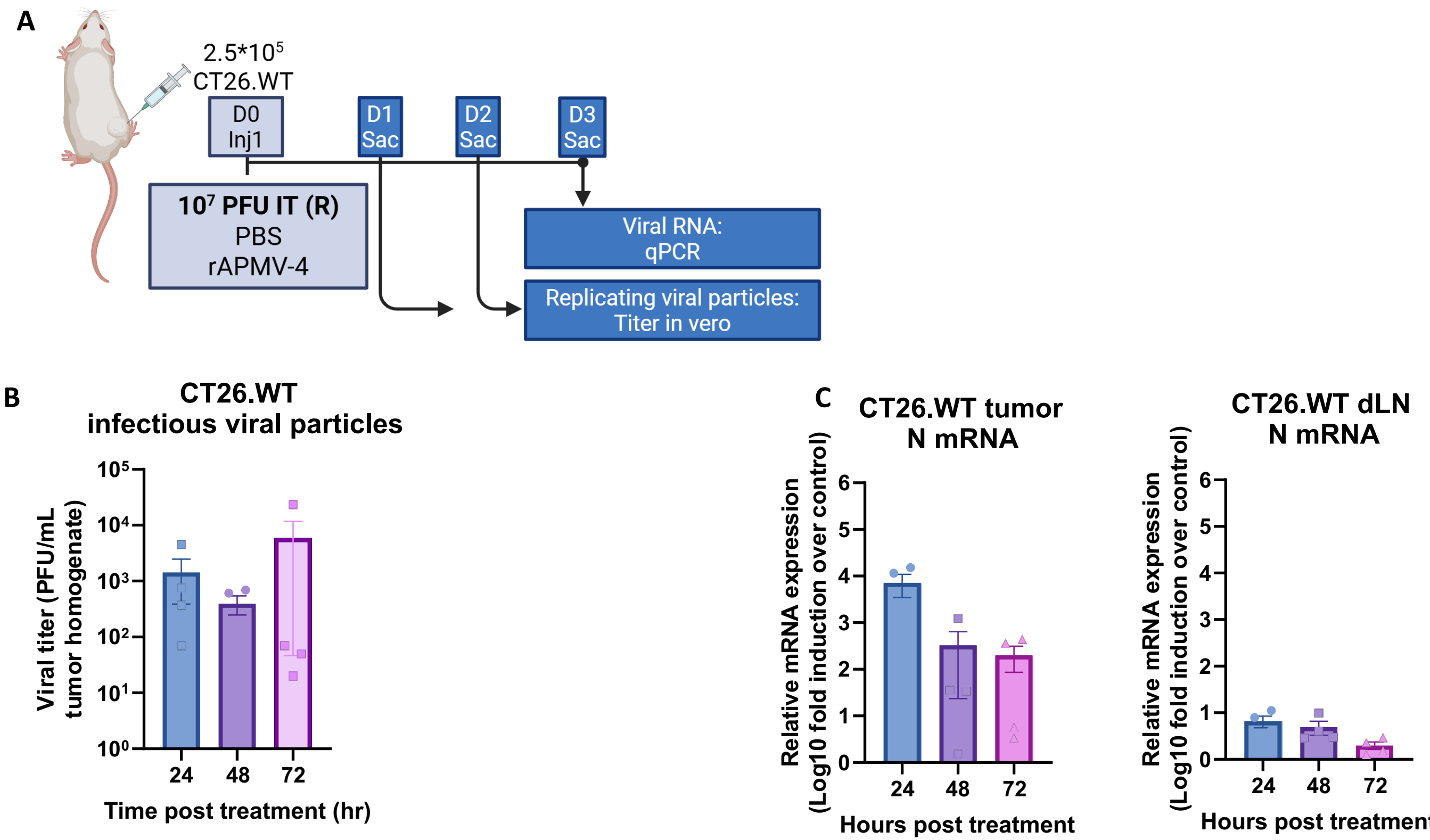


**Figure S14**: **rAPMV-4 *in vivo* replication kinetics. A,** Experimental schematic for **B-C. B,** Infectious viral titers from tumor homogenates, calculated by infection and immunofluorescence of vero cells. **C,** Relative viral mRNA levels in tumors (left) and dLNs (right). N = 4 animals per group.
